# Nitrate Nitrogen and pH Correlate with Changes in Rhizosphere Microbial Community Assemblages during Invasion of Ambrosia artemisiifolia and Bidens pilosa

**DOI:** 10.1128/spectrum.03649-22

**Published:** 2022-12-13

**Authors:** Liting Wang, Qiao Li, Chunying li, Chunpeng Wu, Fengxin Chen, Xue Chen, Fengjuan Zhang

**Affiliations:** a College of Life Science, Hebei University, Baoding, Hebei, China; b State Key Laboratory for Biology of Plant Diseases and Insect Pests, Institute of Plant Protection, Chinese Academy of Agricultural Sciences, Beijing, China; c School of Life Sciences, Fudan University, Yangpu, Shanghai, China

**Keywords:** invasive *Asteraceae* plants, NO_3_-N, rhizosphere soil microbial assemblages, pH

## Abstract

The rhizosphere of invasive plants presumably develops different soil microbial assemblages compared with native plants, which may hinder or promote their invasion. However, to date, no studies have clearly explored rhizosphere microbial community assemblages during invasion. The invasive species Ambrosia artemisiifolia L. and Bidens pilosa L. are widely distributed in China and are known to reduce local biodiversity and cause agricultural losses. Monoculture of A. artemisiifolia or B. pilosa, a mixture of each invasive and native species, and monoculture of native species were established to simulate different degrees of invasion. Metagenomic sequencing techniques were used to test microbial community structure and function. The aim was to explore the drivers of the assembly of peculiar functional microbes in the rhizosphere soil of invasive species during the long-term invasive-native species interaction. Compared with the native species, the relative abundance of 34 microbial genera was higher in the rhizosphere soil of the invasive species. The NO_3_-N concentration in the rhizosphere soil from the *A. artemisiifolia* and *B. pilosa* monocultures was lower than that from monocultures of the three native plants, whereas pH followed the opposite trend. The NO_3_-N concentration was significantly and negatively correlated with *Sporichthya*, *Afipia*, *Actinokineospora*, and *Pseudolabrys*. pH was positively correlated with *Bradyrhizobium*, *Actinoplanes*, *Micromonospora*, *Steroidobacter*, *Burkholderia*, and *Labilithrix*. The differences in soil microbes, NO_3_-N concentrations, and pH between native and invasive species suggest that the rhizosphere soil microbial assemblages may vary. The reduced NO_3_-N concentration and increased pH corelated with changes in rhizosphere microbial community during *A. artemisiifolia* and *B. pilosa* invasion.

**IMPORTANCE** Soil microbial communities play a vital role in the growth of invasive plants. Invasive species may shape peculiar functional microbes in the rhizosphere soil of an invasive species to benefit its growth. However, the drivers of the assembly of soil microbial communities in the rhizosphere soil of invasive species remain unclear. Our study established the relationship between soil microbial communities and soil chemical properties during invasion by *A. artemisiifolia* and *B. pilosa*. Additionally, it showed that the presence of the invasive plants correlated with changes in NO_3_-N and pH, as well as in rhizosphere microbial community assemblage. Furthermore, the study provided important insights into the difference in the microbial community assembly between native and invasive plant species.

## INTRODUCTION

Biological invasion is regarded as the second most substantial threat to biodiversity in terrestrial ecosystems, and it has considerable ecological, economic, and social impacts (1, 2). Soil microbial communities play a vital role in the growth of invasive plants. The rhizospheres of invasive plants are believed to develop different soil microbial assemblages from those that characterize the rhizospheres of native plants, which may hinder or promote the invasion of plant species (3, 4). However, there are few studies focused on the differences in functional microbes within rhizosphere soil between invasive and native species. Furthermore, most of these studies have not clearly reported how the soil microbial community assembles during their invasion (5, 6). Therefore, the assemblages of the soil microbial community and the factors that affect such assemblage remain an important focus for research.

The structure of soil microbial communities can be influenced by various abiotic and biotic factors. Plant species and diversity drive changes in soil microbial communities by altering the diversity and level of soil leachate (7, 8). Temperature, aridity, and soil properties may also affect soil microbial community assemblages (9). Temperature and soil carbon adjust the diversity of soil archaea and aridity, while vegetation attributes and pH regulate the diversity of soil bacteria (10). The compositional similarity of soil microbial communities tends to decrease with increasing geographical distance (11). Alien invasive plant species are thought to have little short-term influence on soil microbial structure. A longer interaction process is required to destroy the long-term equilibrium symbiosis between native plants and soil microorganisms, which has a considerable impact on soil microbial community (12, 13). Most studies have characterized soil biota in samples collected from the field (14, 15). However, these methods cannot accurately determine the duration of invasion. Therefore, the results may have been confounded by heterogeneity in the biotic and abiotic environments. Long-term manipulated field experiments can profit homogeneity and are essential for identifying the factors that drive microbial community assemblages in the rhizosphere soil of invasive species during the invasion process.

Ambrosia artemisiifolia L. and Bidens pilosa L. are two invasive plants of the *Asteraceae* family that are widely distributed in China (16, 17). Their invasion reduces local biodiversity, impairs ecosystem functioning, and results in agricultural losses (18). Soil microbial diversity, such as arbuscular mycorrhizal fungi in the rhizosphere soil of A. artemisiifolia and B. pilosa, has been shown to be different from that of native plants (19). Furthermore, it has been suggested that the soil microbial community supports the invasive plants *A. artemisiifolia* and *B. pilosa* to outcompete the native plants (19). However, the key drivers of the assembly of the peculiar functional microbes in the rhizosphere soil of invasive species during long-term interaction processes remain unknown. To fill these gaps in our knowledge, we conducted long-term homogeneous garden experiments to characterize the rhizosphere microbial communities of native and invasive plants during invasion by *A. artemisiifolia* and *B. pilosa*. We hypothesized that the rhizosphere microbial communities of *A. artemisiifolia* and *B. Pilosa*, as well as the factors that affect the soil microbial assemblages, may be similar to those of *Asteraceae*. We performed a comparative analysis of the effects of different degrees of invasion by *A. artemisiifolia* and *B. pilosa* on the microbial community and the chemical properties of the rhizosphere soil. The relationships between the microbial community and chemical factors were analyzed to identify which factors significantly correlate with changes in the microbial community.

## RESULTS

### Comparative analysis of the effects of different degrees of invasion by *A. artemisiifolia* and *B. pilosa* on the microbial community in the rhizosphere soil.

**FIG 1:**
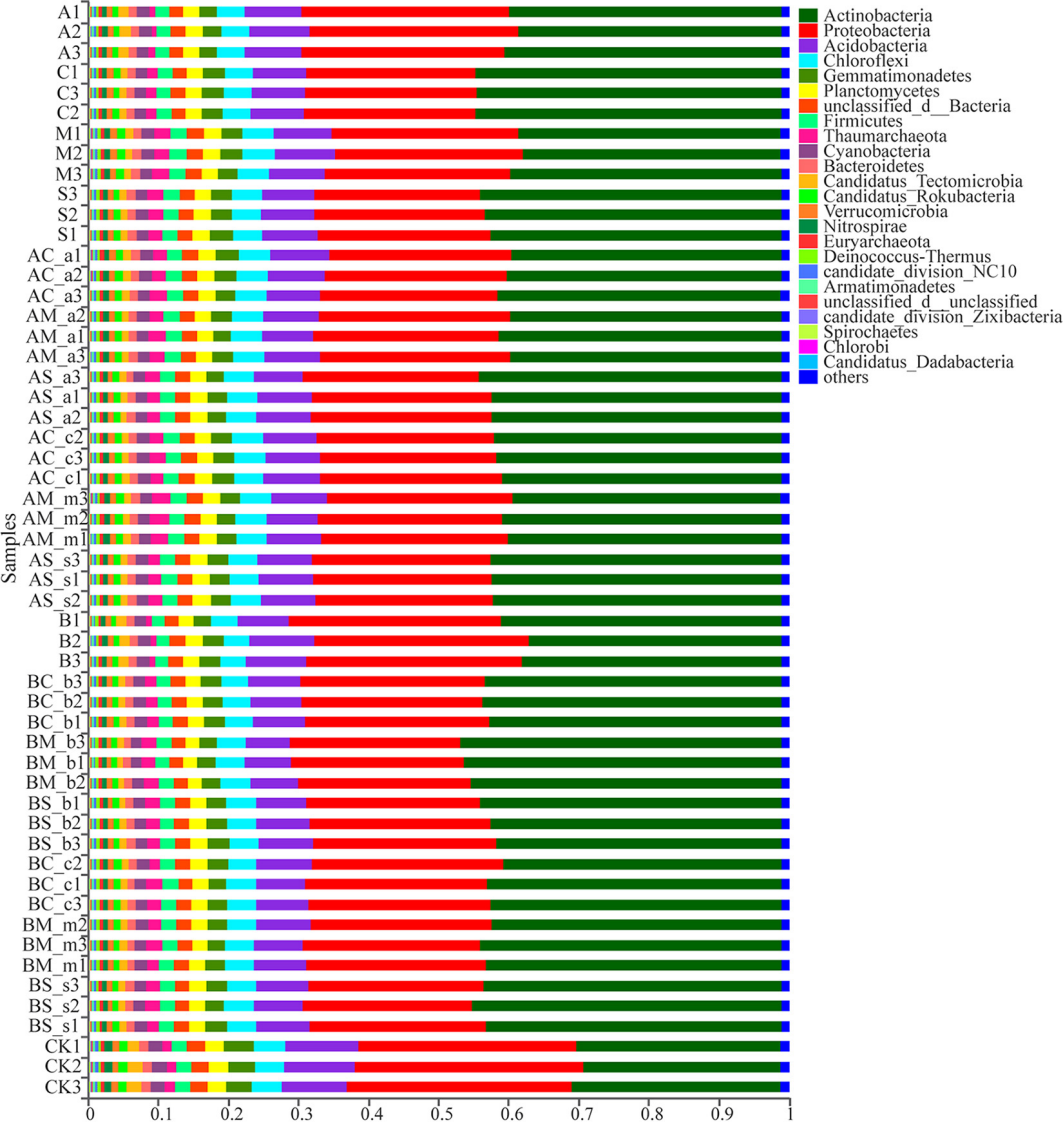
The microbial community of rhizosphere soil for two invasive and three native plants species at phylum level. A, *A. artemisiifolia* monoculture; C, *C. album* monoculture; M, *M. officinalis* monoculture; S, *S. viridis* monoculture; AC-a, *A. artemisiifolia* in the mixture between *A. artemisiifolia* and *C. album*; AM-a, *A. artemisiifolia* in the mixture between *A. artemisiifolia* and *M. officinalis*; AS-a, *A. artemisiifolia* in the mixture between *A. artemisiifolia* and *S. viridis*; AC-c, *C. album* in the mixture between *A. artemisiifolia* and *C. album*; AM-m, *M. officinalis* in the mixture between *A. artemisiifolia* and *M. officinalis*; AS-s, *S. viridis* in the mixture between *A. artemisiifolia* and *S. viridis*; B, *B. pilosa* monoculture; BC-b, *B. pilosa* in the mixture between *B. pilosa* and *C. album*; BM-b, *B. pilosa* in the mixture between *B. pilosa* and *M. officinalis*; BS-b; *B. pilosa* in the mixture between *B. pilosa* and *S. viridis*; BC-c, *C. album* in the mixture between *B. pilosa* and *C. album*; BM-m, *M. officinalis* in the mixture between *B. pilosa* and *M. officinalis*; BS-s, *S. viridis* in the mixture between *B. pilosa* and *S. viridis*; CK, no plant.

**FIG 2:**
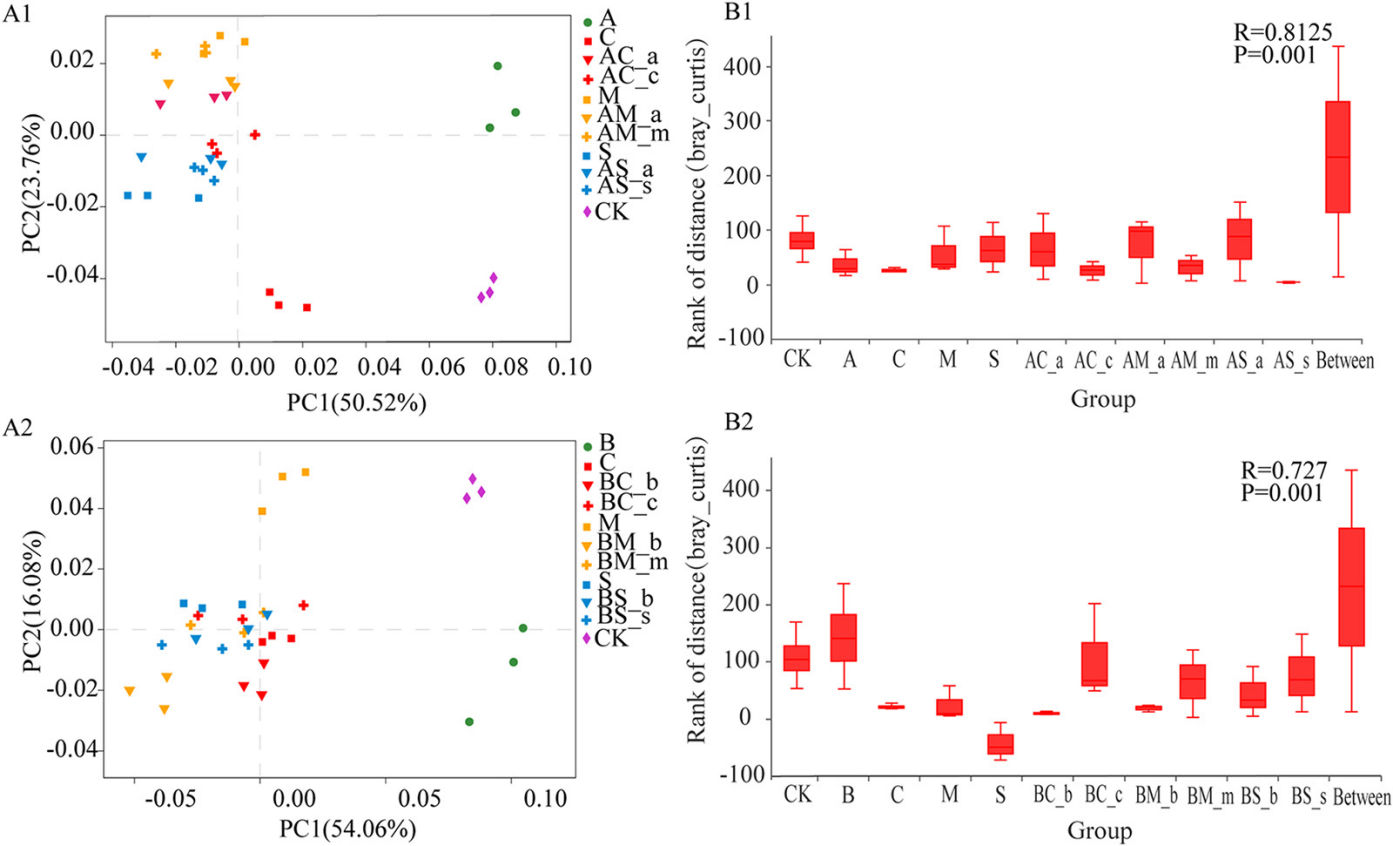
Differences in microbial community of invasive and native species rhizosphere soil under different treatments. (A1) PCoA analysis of microbial community of *A. artemisiifolia* and three native species in the rhizosphere soil of the monoculture and mixture treatments. (A2) PCoA analysis of microbial community of *B. pilosa* and three native species in the rhizosphere soil of the monoculture and mixture treatments. (B1) ANOSIM comparative analysis of similarities in soil microbial community of the rhizosphere soil of between *A. artemisiifolia* and three native species under different treatments. (B2) ANOSIM comparative analysis of similarities in soil microbial community of the rhizosphere soil of between *B. pilosa* and three native species under different treatments. A, *A. artemisiifolia* monoculture; C, *C. album* monoculture; M, *M. officinalis* monoculture; S, *S. viridis* monoculture; AC-a, *A. artemisiifolia* in the mixture between *A. artemisiifolia* and *C. album*; AM-a, *A. artemisiifolia* in the mixture between *A. artemisiifolia* and *M. officinalis*; AS-a, *A. artemisiifolia* in the mixture between *A. artemisiifolia* and *S. viridis*; AC-c, *C. album* in the mixture between *A. artemisiifolia* and *C. album*; AM-m, *M. officinalis* in the mixture between *A. artemisiifolia* and *M. officinalis*; AS-s, *S. viridis* in the mixture between *A. artemisiifolia* and *S. viridis*; B, *B. pilosa* monoculture; BC-b, *B. pilosa* in the mixture between *B. pilosa* and *C. album*; BM-b, *B. pilosa* in the mixture between *B. pilosa* and *M. officinalis*; BS-b; *B. pilosa* in the mixture between *B. pilosa* and *S. viridis*; BC-c, *C. album* in the mixture between *B. pilosa* and *C. album*; BM-m, *M. officinalis* in the mixture between *B. pilosa* and *M. officinalis*, BS-s, *S. viridis* in the mixture between *B. pilosa* and *S. viridis*; CK, no plant.

**FIG 3:**
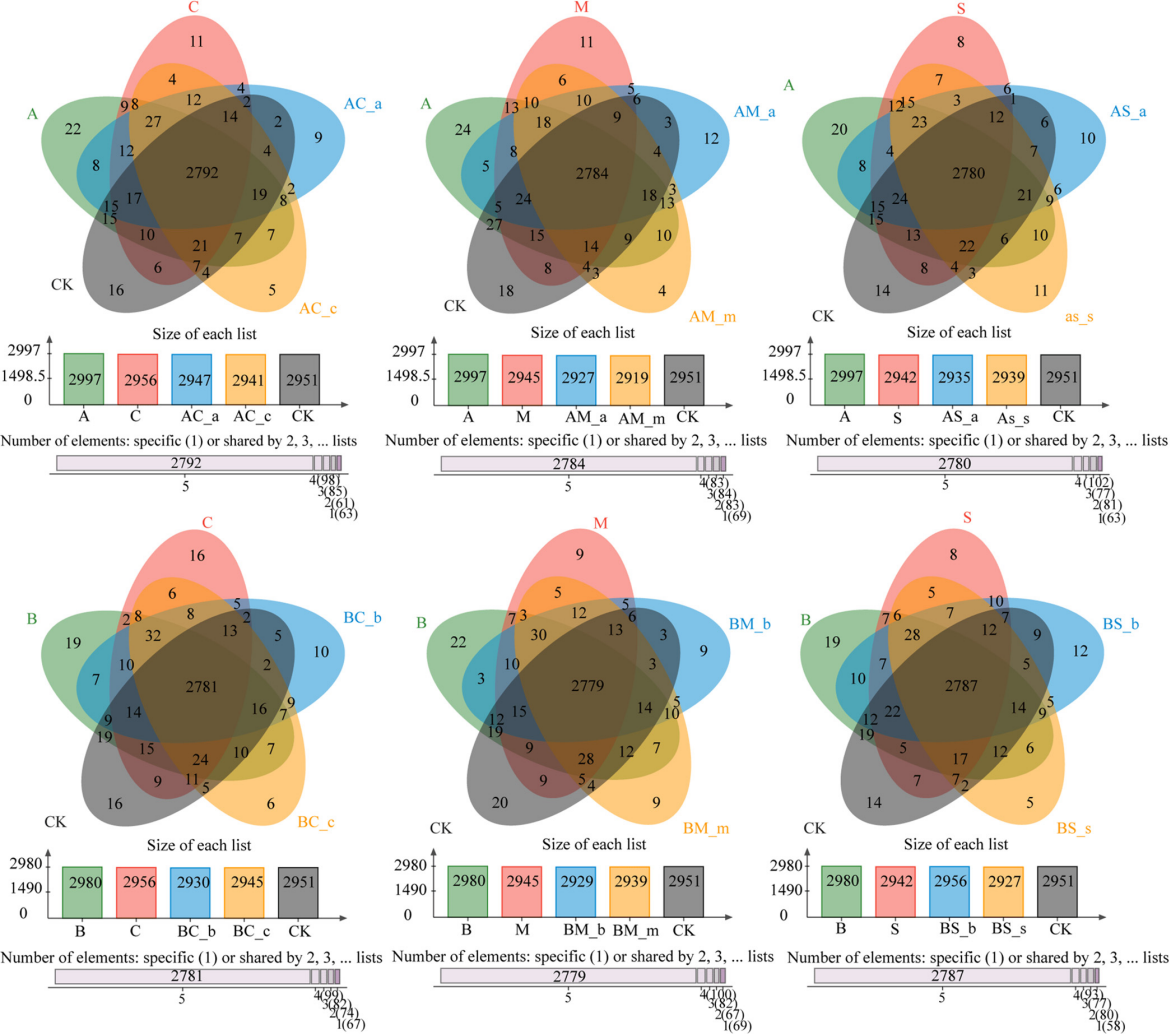
Venn diagram of soil microbial community in the rhizosphere soil of invasive and native plant species at the genus level. The overlapping part represents the species common to multiple sample groups, the nonoverlapping part represents the species unique to the sample group, and the number represents the number of corresponding species. A, *A. artemisiifolia* monoculture, C, *C. album* monoculture; M, *M. officinalis* monoculture; S, *S. viridis* monoculture; AC-a, *A. artemisiifolia* in the mixture between *A. artemisiifolia* and *C. album*; AM-a, *A. artemisiifolia* in the mixture between *A. artemisiifolia* and *M. officinalis*; AS-a, *A. artemisiifolia* in the mixture between *A. artemisiifolia* and *S. viridis*; AC-c, *C. album* in the mixture between *A. artemisiifolia* and *C. album*; AM-m, *M. officinalis* in the mixture between *A. artemisiifolia* and *M. officinalis*; AS-s, *S. viridis* in the mixture between *A. artemisiifolia* and *S. viridis*; B, *B. pilosa* monoculture; BC-b, *B. pilosa* in the mixture between *B. pilosa* and *C. album*; BM-b, *B. pilosa* in the mixture between *B. pilosa* and *M. officinalis*; BS-b; *B. pilosa* in the mixture between *B. pilosa* and *S. viridis*; BC-c, *C. album* in the mixture between *B. pilosa* and *C. album*; BM-m, *M. officinalis* in the mixture between *B. pilosa* and *M. officinalis*; BS-s, *S. viridis* in the mixture between *B. pilosa* and *S. viridis*; CK, No plant.

**FIG 4:**
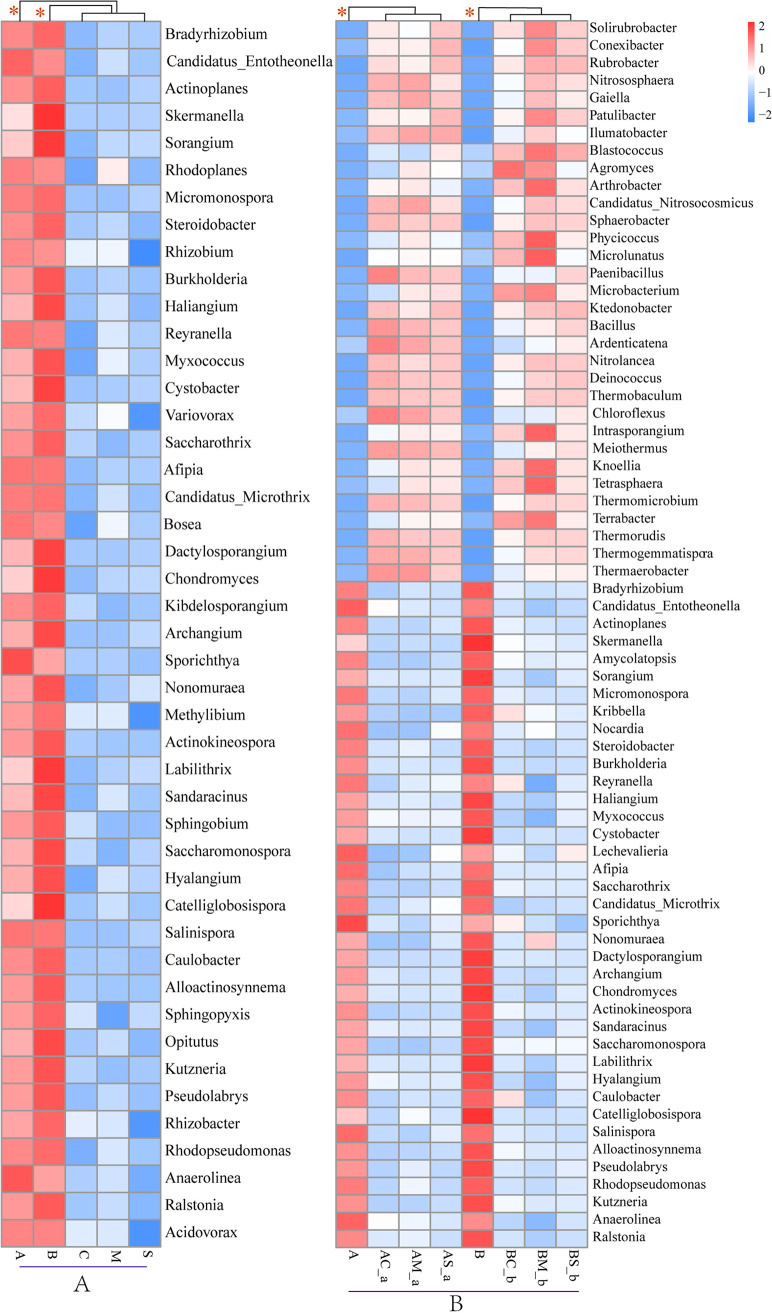
Genera gathered in the rhizosphere soil of *A. artemisiifolia* and *B. pilosa.* (A) The genera whose relative abundances in the rhizosphere soil of the monoculture of *A. artemisiifolia* and *B. pilosa* were higher than that of three native species. (B) The genera whose relative abundances in the rhizosphere soil of the monoculture of *A. artemisiifolia* and *B. pilosa* were higher than that of mixture of of *A. artemisiifolia* and *B. pilosa.* Different color indicated the difference in relative abundance of genera, red color indicates the higher relative abundance of genera, and blue indicates the lower relative abundance of genera. *Significant among the treatments. A, *A. artemisiifolia* monoculture; B, *B. pilosa* monoculture; C, *C. album* monoculture; M, *M. officinalis* monoculture; S, *S. viridis* monoculture; AC-a, *A. artemisiifolia* in the mixture between *A. artemisiifolia* and *C. album*; AM-a, *A. artemisiifolia* in the mixture between *A. artemisiifolia* and *M. officinalis*; AS-a, *A. artemisiifolia* in the mixture between *A. artemisiifolia* and *S. viridis*; BC-b, *B. pilosa* in the mixture between *B. pilosa* and *C. album*; BM-b, *B. pilosa* in the mixture between *B. pilosa* and *M. officinalis*; BS-b; *B. pilosa* in the mixture between *B. pilosa* and *S. viridis*.

The microbial composition of the rhizosphere soil was analyzed at the phylum and genus levels for the two invasive species *A. artemisiifolia* and *B. pilosa* and for the three native plants Chenopodium album L., Melilotus officinalis Ledeb., and Setaria viridis (L.) Beauv. *Actinobacteria*, *Proteobacteria*, *Acidobacteria*, and *Chloroflexi* were the dominant phyla in the rhizosphere soil for all five species (Fig. 1). Principal-component analysis at the genus level showed that significant differences were observed between monoculture and the mixed treatments of *A. artemisiifolia*, although no significant difference between samples (e.g., *A. artemisiifolia* in the mixture between *A. artemisiifolia* and *C. album* [AC-a] and *C. album* in the mixture between *A. artemisiifolia* and *C. album* [AC-c], *A. artemisiifolia* in the mixture between *A. artemisiifolia* and *M. officinalis* [AM-a] and *M. officinalis* in the mixture between *A. artemisiifolia* and *M. officinalis* [AM-m], and *A. artemisiifolia* in the mixture between *A. artemisiifolia* and *S. viridis* [AS-a] and *S. viridis* in the mixture between *A. artemisiifolia* and *S. viridis* [AS-s]) was observed, as their points were mainly closely located in the second or third quadrants (Fig. 2). The number of species in the soil for the two invasive plants was greater than that of the three native plants growing under monoculture. The number of microbial species in the soil for the two invasive plants in the monoculture was greater than that for the invasive and native plants in the mixture treatment (Fig. 3). The relative abundance of bacteria in the rhizosphere soil for *A. artemisiifolia* and *B. pilosa* monocultures was also higher than that in the native species monoculture, while no significant difference was found in the rhizosphere soil of between the invasive and the native species for mixture treatments (Table S1 in the supplemental material). After the relative abundances of genera were compared between the monocultures of two invasive and three native species, and between the monocultures of invasive *A. artemisiifolia* (or *B. pilosa*) and the mixtures of invasive species, respectively, 34 genera, including *Bradyrhizobium*, *Candidatus*, *Entotheonella*, *Skermanella*, *Actinoplanes*, *Sorangium*, *Micromonospora*, *Steroidobacter*, and *Burkholderia*, were found whose relative abundances in the *A. artemisiifolia* and *B. pilosa* monocultures were higher than those in the monocultures of native species or the mixtures of invasive species, respectively (Fig. 4A). Meanwhile, microbial communities recorded in the rhizosphere soil of the two invasive plants were related to companion species in the mixture treatment (Fig. 4B).

### Comparative analysis of the effects of different degrees of invasion by *A. artemisiifolia* and *B. pilosa* on the chemical properties of the rhizosphere soil.

Total nitrogen concentration (TN) in the soil was significantly lower for both *A. artemisiifolia* and *B. pilosa* when the invasive species were grown alone, compared with the soils of most other planting arrangements (Table S2 and S3). The nitrate nitrogen (NO_3_-N) concentration in the two invasive species was lower than that in the three native species planted as monocultures, while the opposite trend was observed for pH. Compared with the two invasive species in the monoculture treatments, the NO_3_-N concentration in the mixed treatments increased significantly, whereas pH decreased. The change in ammonium nitrogen (NH_4_-N) concentration in the invasive species with mixed treatments was significantly associated with the native species that it was planted alongside. Compared with the two invasive species in the monoculture treatment, the NH_4_-N concentration decreased when the species was grown with *C. album* but not when it was grown with *S. viridis* or *M. officinalis*, in which cases it remained unaltered. A similar trend was observed with respect to the changes in nutrient levels and pH in the rhizosphere soil of *A. artemisiifolia* and *B. pilosa* in mixed treatments, which were closely related to the native species (Table S2 and S3). When *A. artemisiifolia* or *B. pilosa* competed with *C. album*, TN concentration and pH increased, whereas available potassium, NH_4_-N, and NO_3_-N concentrations decreased. In turn, when *A. artemisiifolia* or *B. pilosa* competed with *M. officinalis*, TN, available potassium (AK), available phosphorus (AP), organic carbon (OC), and NH_4_-N concentration increased, whereas NO_3_-N concentration and pH decreased. Finally, when *A. artemisiifolia* or *B. pilosa* competed with *S. viridis*, TN and OC concentrations increased, whereas total phosphorus (TP), AK, AP, OC, and NH_4_-N concentrations and pH decreased. TN concentration was higher in the mixed treatments compared with monocultures for all three native species.

### Comparative analysis of the effects of different degrees of invasion by *A. artemisiifolia* and *B. pilosa* on the expression of functional genes related to nitrate synthesis and decomposition in rhizosphere soil.

**FIG 5:**
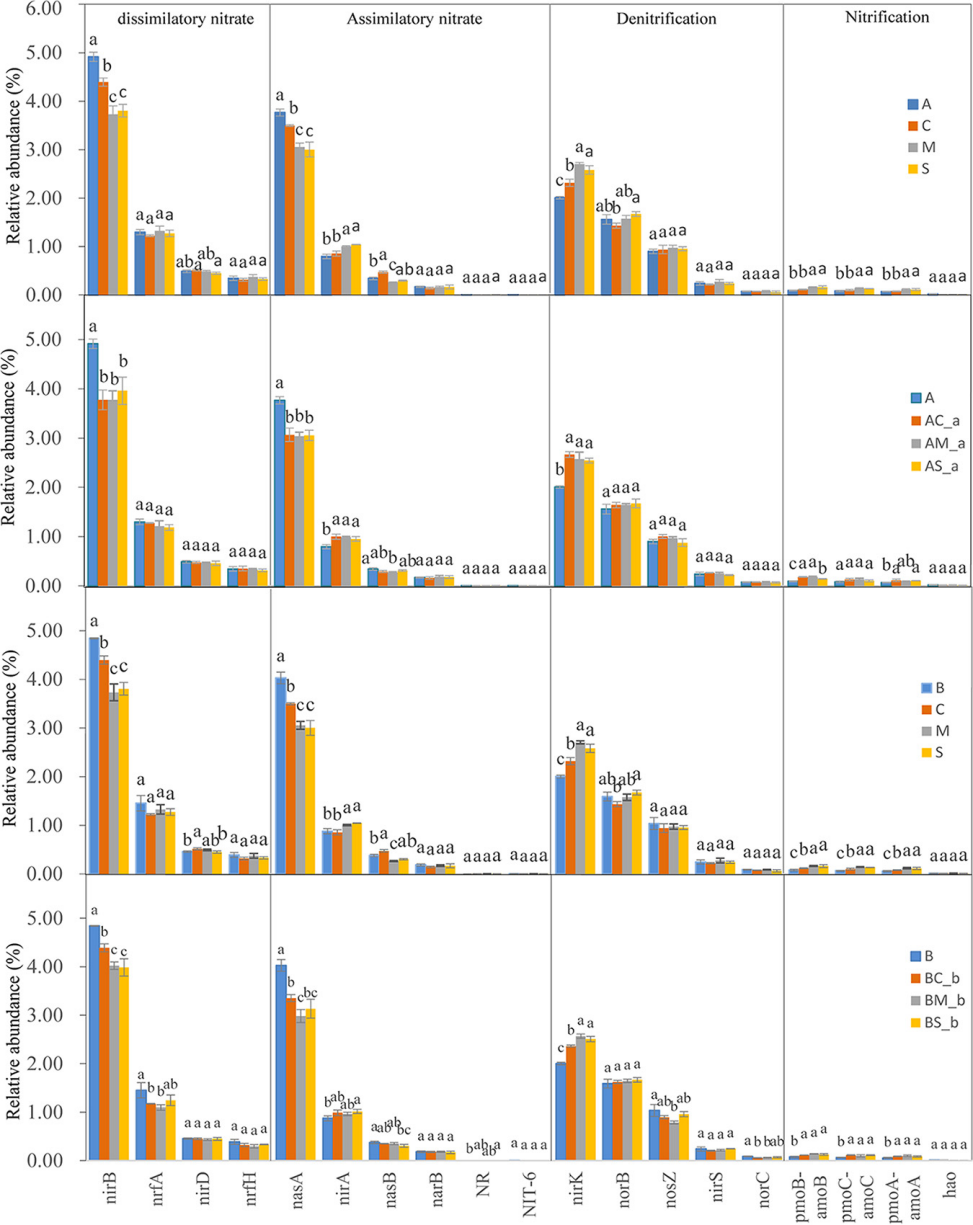
Significant differences in relative abundance of functional genes related to nitrate synthesis and decomposition in two invasive and three native species in different treatments. A, *A. artemisiifolia* monoculture; B, *B. pilosa* monoculture; C, *C. album* monoculture; M, *M. officinalis* monoculture; S, *S. viridis* monoculture; AC-a, *A. artemisiifolia* in the mixture between *A. artemisiifolia* and *C. album*; AM-a, *A. artemisiifolia* in the mixture between *A. artemisiifolia* and *M. officinalis*; AS-a, *A. artemisiifolia* in the mixture between *A. artemisiifolia* and *S. viridis*; BC-b, *B. pilosa* in the mixture between *B. pilosa* and *C. album*; BM-b, *B. pilosa* in the mixture between *B. pilosa* and *M. officinalis*; BS-b; *B. pilosa* in the mixture between *B. pilosa* and *S. viridis.* Different letters in lower case indicate significant differences of four treatments at *P < *0.05.

The relative abundances of genes that were related to dissimilatory nitrate and assimilatory nitrate were higher than those of the denitrification and nitrification within the different degrees of *A. artemisiifolia* and *B. pilosa* invasion tested (Fig. 5 and Fig. S1). The relative abundance of genes that were related with nitrification was lower than that of genes related with denitrification. Overall, our results suggest that the microbial community ability to decompose NO_3_-N was higher than its ability to synthesize NO_3_-N.

The relative abundances of the *nirB* and *nasA* genes were the highest among all the genes regarding dissimilatory nitrate reduction and assimilatory nitrate reduction. The relative abundances of the *nirB* and *nasA* genes isolated from the soils of the two invasive species were higher under monoculture than under mixed treatments. This trend was also observed when comparing the invasive and native monoculture treatments, whereby *nirB* and *nasA* relative abundances were highest in the invasive monoculture treatments. As for the nitrification ability of the soil microorganisms, the changes in functional genes such as *pmoB-amoB*, *pmoC-amoC*, and *pmoA-amoA* were similar. The relative abundances of *pmoB-amoB*, *pmoC-amoC*, and *pmoA-amoA* were lower in invasive species monocultures, compared with mixed treatments or native species monocultures.

### Comparative analysis of the effects of different degrees of invasion by *A. artemisiifolia* and *B. pilosa* on plant nitrogen-absorption capacity.

**FIG 6:**
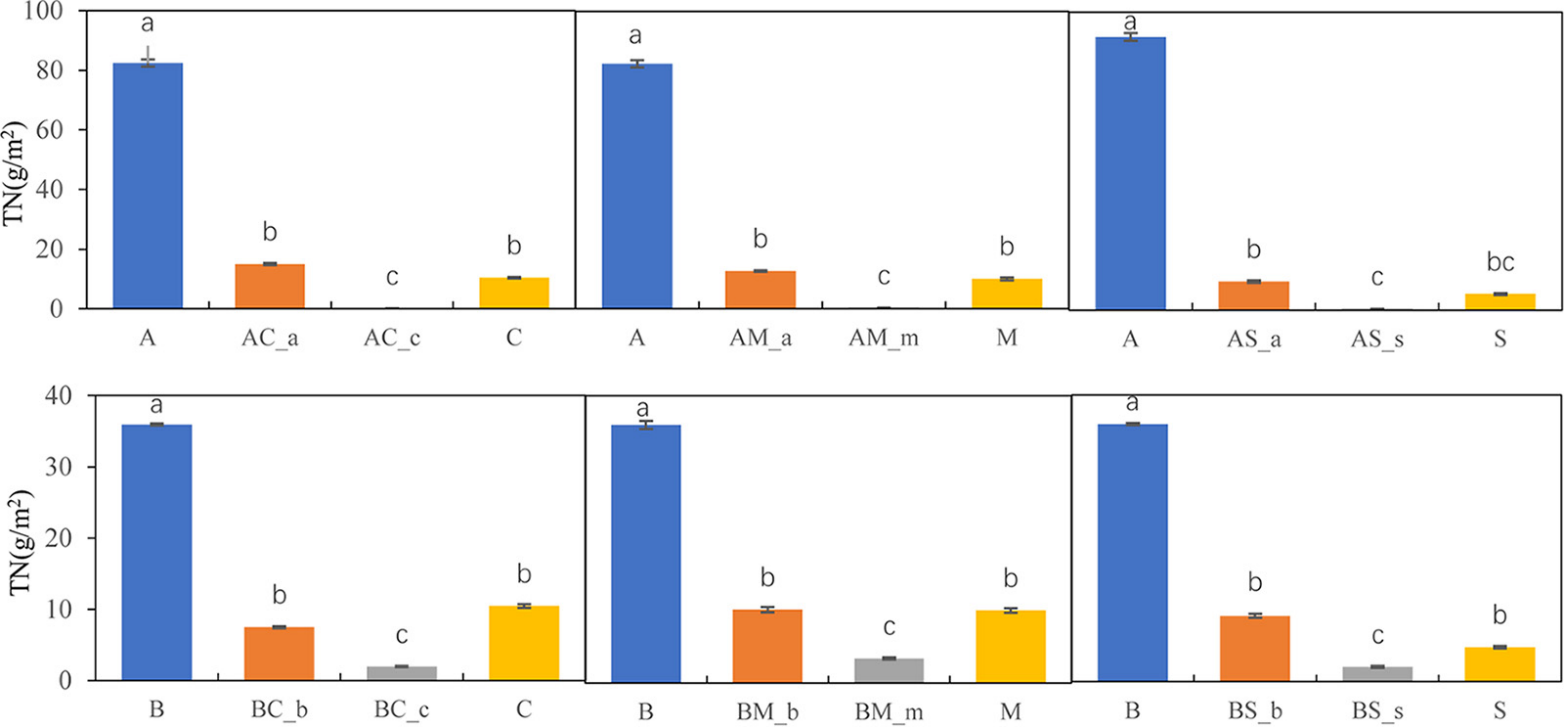
The total nitrogen content per m^2^ in different treatments. A, *A. artemisiifolia* monoculture; C, *C. album* monoculture; M, *M. officinalis* monoculture; S, *S. viridis* monoculture; AC-a, *A. artemisiifolia* in the mixture between *A. artemisiifolia* and *C. album*; AM-a, *A. artemisiifolia* in the mixture between *A. artemisiifolia* and *M. officinalis*; AS-a, *A. artemisiifolia* in the mixture between *A. artemisiifolia* and *S. viridis*; AC-c, *C. album* in the mixture between *A. artemisiifolia* and *C. album*; AM-m, *M. officinalis* in the mixture between *A. artemisiifolia* and *M. officinalis*; AS-s, *S. viridis* in the mixture between *A. artemisiifolia* and *S. viridis*; B, *B. pilosa* monoculture; BC-b, *B. pilosa* in the mixture between *B. pilosa* and *C. album*; BM-b, *B. pilosa* in the mixture between *B. pilosa* and *M. officinalis*; BS-b; *B. pilosa* in the mixture between *B. pilosa* and *S. viridis*; BC-c, *C. album* in the mixture between *B. pilosa* and *C. album*; BM-m: *M. officinalis* in the mixture between *B. pilosa* and *M. officinalis*; BS-s: *S. viridis* in the mixture between *B. pilosa* and *S. viridis*; Different lowercase letters indicate significant differences of four treatments at *P < *0.05.

Nitrogen concentration was highest in the two invasive species planted as monocultures, compared with the rest of the treatments. A similar trend was observed for TN uptake per unit area, in which case, the highest TN uptake was observed in the invasive species monocultures (Fig. 6).

### Relationships between microbial community and chemical factors in the rhizosphere soil.

**FIG 7:**
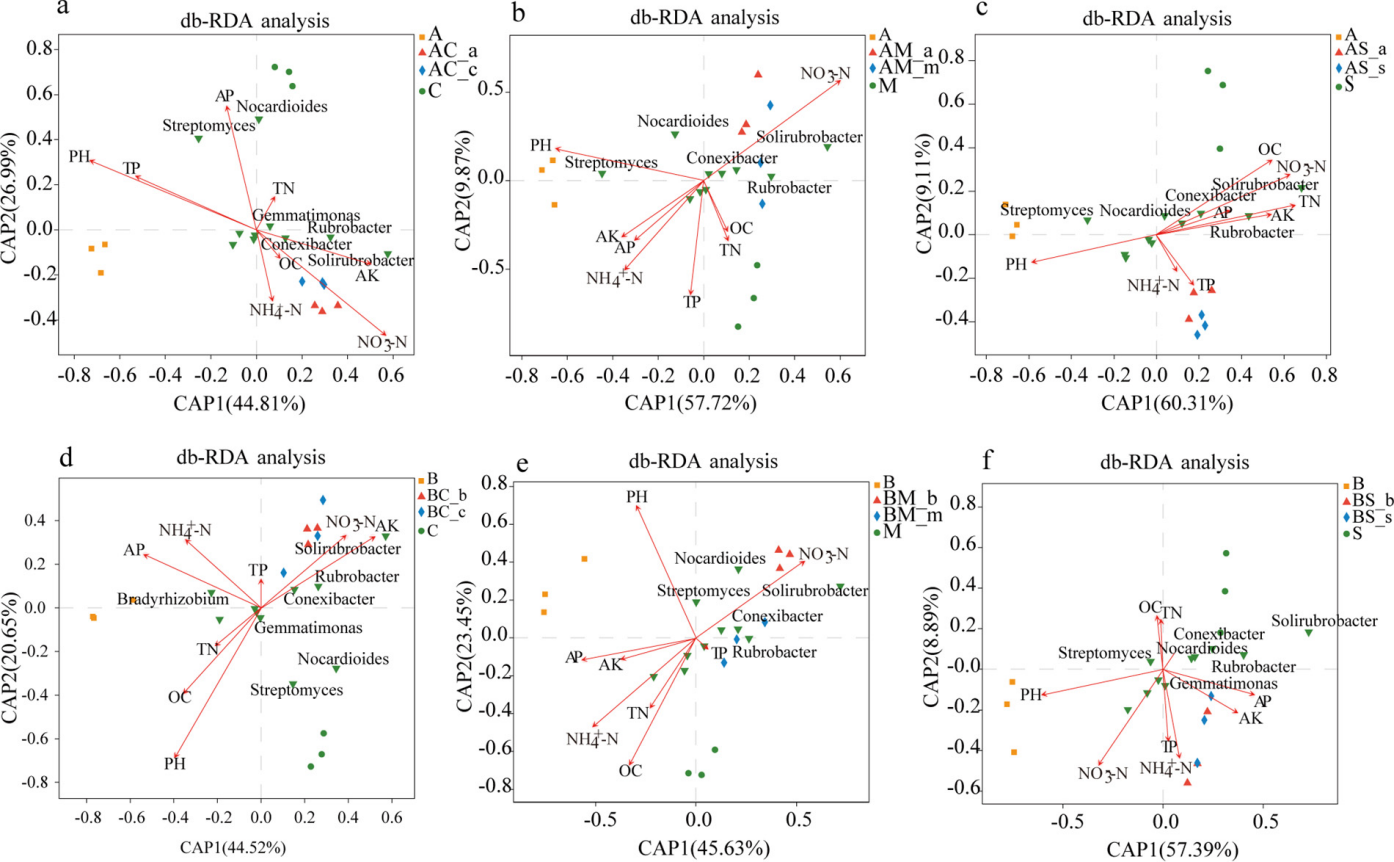
db-RDA analysis of rhizosphere soil microorganisms and soil physicochemical factors of two invasive and three native plants under different treatments. The arrows in the figure represent soil physicochemical indicators, the green inverted triangles represent high abundance microbial genera, and the other graphs represent soil samples. The length of the arrows represents the degree of correlation between the measured soil physicochemical factors and the rhizosphere soil microbial community and microbial species distribution. The longer the line, the higher the degree of correlation, and vice versa; the size of the included angle represents the positive and negative correlation. Right angles are represented as having no correlation. A, *A. artemisiifolia* monoculture; C, *C. album* monoculture; M, *M. officinalis* monoculture; S, *S. viridis* monoculture; AC-a, *A. artemisiifolia* in the mixture between *A. artemisiifolia* and *C. album*; AM-a, *A. artemisiifolia* in the mixture between *A. artemisiifolia* and *M. officinalis*; AS-a, *A. artemisiifolia* in the mixture between *A. artemisiifolia* and *S. viridis*; AC-c, *C. album* in the mixture between *A. artemisiifolia* and *C. album*; AM-m, *M. officinalis* in the mixture between *A. artemisiifolia* and *M. officinalis*; AS-s, *S. viridis* in the mixture between *A. artemisiifolia* and *S. viridis*; B, *B. pilosa* monoculture; BC-b, *B. pilosa* in the mixture between *B. pilosa* and *C. album*; BM-b, *B. pilosa* in the mixture between *B. pilosa* and *M. officinalis*; BS-b; *B. pilosa* in the mixture between *B. pilosa* and *S. viridis*; BC-c, *C. album* in the mixture between *B. pilosa* and *C. album*; BM-m, *M. officinalis* in the mixture between *B. pilosa* and *M. officinalis*; BS-s, *S. viridis* in the mixture between *B. pilosa* and *S. viridis*; AK, available potassium; AP, available phosphorus; OC, organic carbon; NH_4_-N, ammonium nitrogen; NO_3_-N, nitrate nitrogen; TP, total phosphorus; TN, total nitrogen concentration.

The pH and NO_3_-N concentration were two factors highly correlated with changes in the microbial community in the rhizosphere soil of *A. artemisiifolia* and *B. pilosa* (Fig. 7 and Table S4). Furthermore, pH was positively correlated with *Bradyrhizobium*, *Actinoplanes*, *Micromonospora*, *Steroidobacter*, *Burkholderia*, *Dactylosporangium*, *Labilithrix*, *Salinispora*, and *Alloactinosynnema*, while NO_3_-N was positively correlated with *Bradyrhizobium*, *Micromonospora*, *Sporichthya*, *Afipia*, *Actinokineospora*, *Salinispora*, and *Pseudolabrys* during the various degrees of *A. artemisiifolia* invasion (Fig. S2). Significant correlations were defined at *P < *0.05. In turn, pH was positively correlated with *Bradyrhizobium*, *Actinoplanes*, *Sorangium*, *Micromonospora*, *Steroidobacter*, *Burkholderia*, *Haliangium*, *Chondromyces*, *Labilithrix*, and *Sandaracinus*, whereas NO_3_-N concentration was positively correlated with *Actinoplanes*, *Afipia*, *Sporichthya*, *Actinokineospora*, *Alloactinosynnema*, *Bradyrhizobium*, *Micromonospora*, and *Pseudolabrys* during different degrees of *B. pilosa* invasion. Overall, pH of the invasive species was positively correlated with *Bradyrhizobium*, *Actinoplanes*, *Micromonospora*, *Steroidobacter*, *Burkholderia*, and *Labilithrix*, and NO_3_-N concentration was positively correlated with *Sporichthya*, *Afipia*, *Actinokineospora*, *Bradyrhizobium*, *Micromonospora*, and *Pseudolabrys.*

## DISCUSSION

Increasing evidence shows that invasive species shape different rhizosphere microbial communities compared with native species (5, 6, 20, 21). Thus, for example, Li et al. (22) suggested that the invasion of *Ageratina adenophora* resulted in the selection of specialized bacterial communities in soils, thereby switching the microbial composition from *Aeromicrobium* and *Marmoricola* to *Reyranella* and *Bradyrhizobium* in the bulk soils and from *Gp4*, *Pirellula*, *Lysobacter*, and *Aridibacterrae* to *Reyranella* and *Streptomyces* in the rhizosphere soils. Similarly, our results demonstrated that specific functional microbes are recruited in the rhizosphere of invasive species, such as *Bradyrhizobium*, *Skeroidobacter*, and *Actinoplanes*, most of which belong to beneficial groups: *Steroidobacter* has denitrification effect (23, 24), and *Actinoplanes* can inhibit the growth of plant pathogens and produce antifungal compounds as well as accelerate nutrient absorption and positively influence plant growth (25, 26). In turn, *Micromonospora*, *Burkholderiales*, and *Anaerolinea* are related to the N and C cycles (27–29), and *Saccharothrix* can generate chitinase and degrade the structure of plant-pathogenic fungi (30). For their part, genera such as *Rhodopseudomonas*, *Actinoplanes*, *Burkholderia*, and Variovorax paradoxus, which are present in the rhizosphere soil, can help Panax notoginseng resist the invasion of root rot disease (31). Consistently, the results reported herein highlighted the fact that the increased relative abundance of *Bradyrhizobium*, *Micromonospora*, *Burkholderiales*, and *Anaerolinea* may enhance the N and C cycles, thereby providing more available nutrients to invasive species. The increased relative abundance of *Rhodopseudomonas* and *Saccharothrix* may benefit the growth of the two invasive species by controlling pests, diseases, and pathogens in the rhizosphere soil. The association between beneficial bacteria and the growth of *A. artemisiifolia* and *B. pilosa* requires further exploration.

Both soil characteristics and plant species play a significant role in shaping rhizosphere microbial communities (32, 33). Various abiotic factors such as pH, nutrient levels and soil texture, and plant characteristics, including biomass production, litter quality, and root exudates, can affect the assembly of soil microbial communities (33, 34). In this study, the long-term homogeneous garden experiment showed that the invasion of *A. artemisiifolia* and *B. pilosa* may alter biotic and abiotic properties of the soil. The relative abundances of 34 genera in the monocultures of *A. artemisiifolia* and *B. pilosa* were higher than those in the monocultured native species and in the mixed treatments of invasive species monocultures, respectively. However, compared with the native species monocultures, NO_3_-N concentration decreased and pH increased in the invasive species monocultures. Differences such as those observed in our study in soil microbial communities and the soil properties may be based on quantitative and qualitative differences in rhizodeposits and the litter quality between invasive and native plant communities (21). Long-term interactions between plants and soil microbial communities may lead to changes in the soil properties (35, 36) that can subsequently affect the growth of microbial communities and plants (10). We suggest that the similar changes in microbial community, pH, and NO_3_-N between *A. artemisiifolia* and *B. pilosa* indicate that they may share a similar mechanism at play in the assembly of rhizosphere microbial communities.

Soil pH has a strong effect on the diversity and structure of soil bacterial communities (37). Soil bacteria grow well in neutral or alkaline environments, with few microbial communities present in acidic soils (38). Different soil microorganisms respond differently to pH, and small pH changes can lead to the screening and enrichment of specific microorganisms (39). Compared with the rhizosphere soil of native species, the increased pH in alkaline environments in the rhizosphere soil of *A. artemisiifolia* and *B. pilosa* benefited bacterial growth, such as *Bradyrhizobium*, *Micromonospora*, *Burkholderia*, *Steroidobacter*, and *Actinoplanes.* Fan et al. (40) suggested that soil pH may play an essential role in the interaction and assemblage processes of the diazotrophic community in the rhizosphere. Similarly, Li et al. (22) suggested that soil pH plays an important role in shaping microbial communities in both rhizosphere and bulk soils of Ageratina adenophora. The invasion of exotic plant species has been shown to increase soil pH (41–43), which is consistent with the results of this study. Specifically, the db_RDA redundancy analysis between the microbial community and the chemical factors in various soil treatments showed that pH was highly correlated with changes in the microbial community in the rhizosphere soil of *A. artemisiifolia* and *B. Pilosa*, suggesting that pH is an important factor influencing the assemblage processes of the microbial community in the rhizosphere soil of *A. artemisiifolia* and *B. pilosa*. Soil pH likely plays a vital role in controlling the interactions and assemblage processes of soil microbial communities (40). However, different plant species may exhibit a preference for acidic, alkaline, or neutral pH conditions to form stable microbial communities to benefit their growth. A neutral pH in the rhizosphere soil of wheat may indicate a more stable diazotrophic community, whereas, for the two alien invasive species *A. artemisiifolia* and *B. pilosa*, soils with alkaline pH may indicate a more stable microbial community.

The invasion of alien plant species may also alter the N cycle of the rhizosphere soil and result in the increase or decrease of NO_3_-N concentration (22, 44). Consistent with the results of previous studies (45, 46), here, we found an association between the presence of invasive species and the lower NO_3_-N concentration in the soil. The decrease in NO_3_-N concentration in the rhizosphere soil may depend on a strong plant N-uptake capacity and changes in the N conversion rate of rhizosphere soils. The higher N-uptake capacity of *A. artemisiifolia* and *B. pilosa*, compared with that of the native species, may lead to a decrease in NO_3_-N concentration in the rhizosphere soil of the invasive species. The uptake of nitrate may be effective in increasing the subsurface soil pH through rhizosphere alkalization, which may lead to differences in N-use efficiency and asymmetrical competition between alien and native plants (47–49). Soil microbial mediation plays a vital role in soil N cycling (50, 51). The circular transformation of N in nature includes four basic processes, namely, fixation, nitrification, ammonification, and denitrification. In our study, we observed a higher relative abundance of genes related to dissimilatory and assimilatory nitrate as well as a lower relative abundance of genes related to denitrification. This may result in lower NO_3_-N concentrations in *A. artemisiifolia* and *B. pilosa* soil*s.* The relative abundance of soil N-cycle functional genes can indirectly reflect the rate of the soil N cycle (52, 53). The relative abundance of the functional genes involved in nitrification (*pmoB-amoB*, *pmoC-amoC*, and *pmoA-amoA*) in the *A. artemisiifolia* and *B. pilosa* monocultures was lower than that observed in the monocultures of native species, suggesting that the invasion of *A. artemisiifolia* and *B. pilosa* reduced the potential nitrification rate of rhizosphere soils. However, the relative abundance of functional genes associated with nitrate decomposition (e.g., *nirB* and *nasA*) was higher than that observed in the monocultures of the native species, suggesting that the long-term invasion of *A. artemisiifolia* and *B. pilosa* may significantly increase the nitrate decomposition in rhizosphere soil and accelerate the decomposition of nitrate, thus leading to a decrease in NO_3_-N concentration in the rhizosphere soil. The invasion of exotic plant species may enhance the soil nitrification rate (54–56), while consistently, the enrichment of NO_3_-N and NH_4_^+^-N in the rhizosphere soil has been observed for alien plant species (51, 57). These differences may be related to secondary metabolites released by alien plant species (58). Additionally, we found that the NO_3_-N concentration was positively correlated with *Sporichthya*, *Afipia*, *Actinokineospora*, *Bradyrhizobium*, *Micromonospor*, and *Pseudolabrys*, suggesting that NO_3_-N is another important factor that influences the assemblage processes of the microbial community in the rhizosphere soil of *A. artemisiifolia* and *B. pilosa*.

## MATERIALS AND METHODS

### Study site.

To study the interaction between invasive plants and soil microorganisms during the invasion process, a long-term field experiment was conducted from 2008 until 2019 at the Langfang Experimental Station of the Chinese Academy of Agricultural Science, Beijing, China (39°30′42″N, 116°36′07″E). The experimental site has a temperate northern climate. The soils at the site are sandy-clay loam ferric Acrisols (FAO/UNESCO classification method). Mean annual precipitation and temperatures for the period from 2008 to 2019 are listed in Table S5.

### Experiment design.

Chenopodium album, *M. officinalis*, and *S. viridis* are widely distributed in farmlands, roadsides, and wastelands and are often found in areas invaded by *A. artemisiifolia* and *B. pilosa*. Experimental plots (3 by 2 m) were set up in May 2008, and 12 treatments were listed to simulate different degrees of invasion: uninvaded, 60% invaded, and 100% invaded areas. They were as follows: (i) uninvaded area included three treatments: monoculture of *C. album* (C), monoculture of *M. officinalis* (M), and monoculture of *S. viridis* (S); (ii) 60% invaded area included six treatments: mixture of *A. artemisiifolia* and *M. officinalis* (AM), mixture of *A. artemisiifolia* and *S. viridis* (AS), mixture of *B. pilosa* and *C. album* (BC), mixture of *B. pilosa* and *M. officinalis* (BM), mixture of *B. pilosa* and *S. viridis* (BS), and mixture of *A. artemisiifolia* and *C. album* (AC); the invasive and native species cover was 60% and 40%, respectively, when grown under competition of invasive species; and (iii) 100% invaded area included two treatments: monoculture of *A. artemisiifolia* (A) and monoculture of *B. pilosa* (B). The invasive species cover was 100%. No plant plots were regarded as a control treatment (Table S6). A total of 100 seeds of invasive and native species were sown in each plot, with 6 replicate plots per treatment. Seventy-two plots were arranged in a randomized complete block design. No fertilizer was applied during the experiment. The plant species composition in each plot was kept constant by manually removing weeds after the seeds germinated during the growing season. Withered plants were pulled out before spring. Invasive and native species were reseeded in their monoculture or in a mixture the following spring. Each plot was irrigated once and field capacity reached 40% before seed germination in spring. Annual rainfall is listed in Table S1. A 1-m-wide buffer strip was established to separate the plots. Samples from each treatment group were collected in 2019.

### Plant and soil sampling.

In the field experiment, growths of the invasive and native plants were not uniform under individual treatments. Initially, there were six replicates for each treatment, but this was later reduced to three, where only plants with similar growth and vigor were used. Huber (59) highlighted that, by identifying the outliers and thinned samples, the individual sample size would more robustly represent the sample mean. Therefore, 3 replicates were included for each treatment, and a total of 54 rhizosphere soil samples of invasive and native species were collected in September 2019. In the treatments with mixed species, rhizosphere soil samples were collected from both native and invasive plants. The rhizosphere soils were collected and preprocessed according to the method described by Tang et al. (60). Specifically, rhizosphere soil was operationally defined as soil adhering to the total roots after gentle shaking. Whole plants with their roots were extracted from the soil and after shaking off the loosely adhering soil, the tightly adhering rhizosphere soil was carefully collected. The soil samples were sieved (<2 mm), and approximately 5 g of soil was stored in a −80°C ultrafreezer for soil metagenomic assays. The remaining soil samples were air dried at 20 to 25°C to determine the soil physicochemical indicators.

### Structural and functional analysis of rhizosphere soil-microbial communities.

**(i) DNA extraction, library construction, and metagenomic sequencing.** Microbial community structure and function in the rhizosphere soil were determined using metagenomic sequencing techniques. Total genomic DNA was extracted from 0.5-g soil samples using the FastDNA Spin kit for Soil (Mpbio, CA, USA) according to the manufacturer’s instructions. The concentration and purity of the extracted DNA were tested using TBS-380 and NanoDrop 2000, respectively. The quality of the DNA extract was checked on a 1% agarose gel. DNA was fragmented to approximately 400 bp using Covaris M220 (Gene Company Limited, China) for paired-end library construction. The paired-end library was built using NEXTFLEX Rapid DNA-Seq (Bio Scientific, Austin, TX, USA). Adapters that include the full complement of the sequencing primer hybridization sites were ligated to the blunt ends of the fragments. Paired-end sequencing was performed on Illumina NovaSeq (Illumina Inc., San Diego, CA, USA) at Majorbio Bio-Pharm Technology Co., Ltd. (Shanghai, China) using NovaSeq Reagent Kits according to the manufacturer’s instructions (www.illumina.com). Sequence data associated with this project were deposited in the NCBI Short Read Archive database (accession number PRJNA899702).

**(ii) Sequence quality control and genome assembly.** Raw reads from metagenome sequencing were used to generate clean reads after removing the adaptor sequences and trimming and removing low-quality reads using fastp (https://github.com/OpenGene/fastp, version 0.20.0) on the free online Majorbio Cloud Platform (cloud.majorbio.com). High-quality reads met the criteria of reads with N bases, a minimum length threshold of 50 bp, and a minimum quality threshold of 20. These high-quality reads were then assembled into contigs using MEGAHIT (parameters: kmer_min = 47, kmer_max = 97, step = 10) (https://github.com/voutcn/megahit, version 1.1.2), which uses succinct de Bruijn graphs.

**(iii) Gene prediction, taxonomy, and functional annotation.** Open reading frames (ORFs) in the contigs were identified using MetaGene (https://www.metagene.de/). Predicted ORFs with a length of over 100 bp were retrieved and translated into amino acid sequences using the NCBI translation table. A nonredundant gene catalog was constructed using CD-HIT (https://packages.debian.org/sid/soapaligner, version 4.6.1) with 90% sequence identity and 90% coverage. After quality control, reads were mapped to the nonredundant gene catalog with 95% identity using SOAPaligner (http://soap.genomics.org.cn/, version 2.21), and gene abundance in each sample was analyzed. Representative sequences of nonredundant gene catalogs were compared with the NCBI NR database with an e-value cutoff of 1e^−5^ using Diamond (https://github.com/bbuchfink/diamond, version 0.8.35) for taxonomic annotation. The Kyoto Encyclopedia of Genes and Genomes (KEGG) annotation was performed using Diamond (https://github.com/bbuchfink/diamond, version 0.8.35) against the KEGG database (http://www.genome.jp/kegg/) with an e-value cutoff of 1e^−5^.

**(iv) Comparative analysis of the effects of different degrees of invasion on the expression of functional genes related to nitrate synthesis and decomposition in rhizosphere soil.**The relative abundances of the functional genes related to the dissimilatory nitrate (*nirB*, *nrfA*, *nirD*, and *nrfH*), assimilatory nitrate (*NasA*, *nirA*, *narB*, *NR*, and *NTT-6*), denitrification (*NirK*, *norB*, *nosZ*, *nirS*, and *norC*), and nitrification (*pmoB-amoB*, *pmoC-amoC*, *pmoA-amoA*, and *hao*) under different treatments were analyzed. The dissimilatory nitrate, assimilatory nitrate, and denitrification were used to compare the difference in NO_3_-N decreases, and nitrification was used to compare the difference in NO_3_-N increases among treatments.

**(v) Soil chemical properties.** Soil samples were air dried and sieved (1-mm mesh) before determination of soil properties. Soil pH was measured using a pH meter (E20-FiveEasyTM pH, Mettler Toledo, Germany) in a 1:5 (fresh soil: deionized water [wt/vol]) suspension after shaking for 30 min. Organic carbon was analyzed using spectrophotometric measurement after digestion with a K_2_Cr_2_O_7_-H_2_SO_4_-soil mixture in a commercial microwave oven. After extraction with sodium bicarbonate, available soil P (AP) was determined using the molybdenum blue method (61). Concentrations of ammonium nitrogen (NH_4_-N) and nitrate nitrogen (NO_3_-N) were analyzed using a SmartChem automatic discontinuous chemical analyzer (62). Total nitrogen (TN) and total phosphorus (TP) were determined using the Kjeldahl and the perchloric acid, and sulfuric acid methods, respectively (63).

**(vi) Nitrogen uptake ability.** For each treatment, a 50 by 50-cm area was selected for each plot. To eliminate edge effects, a 50 by 50-cm area was selected at the center of a 2 by 1-m area within each 3 by 2-m plot. The entire plant, including the aboveground components and the root biomass in the 50 by 50-cm plots was collected, washed to remove any soil particles, and then oven dried at 80°C for 48 h to collect data on dry biomass. Plant biomass per unit area was then obtained. Approximately 2 g of leaves were analyzed for TN concentration, which was measured using the micro-Kjeldahl method (64). The per unit N content of the plant was then calculated using the following formula:

per unit N content of plant (g/kg) = per unit biomass (kg) × N concentration (g/kg)

### Data analysis.

A comparative analysis of the microbial community between the invasive and the native species in the rhizosphere soil under different treatments was performed using the base R package “VennDiagram, vegan, and ggplot2.” The Venn chart indicates the similarity and overlap of the species composition among samples, and the bar chart indicates the species composition of the different groups. Principal coordinates analysis (PCoA) was performed using the prcomp function from the R package “stats” with the Bray-Curtis metric. The Bray-Curtis metric was calculated using the vegdist function from the R package “vegan” (v.2.4-4). The multivariate null hypotheses “no differences among prior defined groups” were examined using nonparametric test ANOSIM (an analog of univariate ANOVA). To statistically support the visual clustering of the rhizosphere soil microorganisms in the principal-component analysis, two invasive plants in the monoculture, three native plants in the monoculture, as well as the invasive and native plants in the mixture were compared using the Wilcoxon rank-sum test followed by the R package “vegan” to draw the Heapmap, which is used to distinguish the relative abundance at the genus level. KEGG pathway enrichment analysis was performed using the function phyper in the R package “stats.” One-way ANOVA was carried out to determine the differences in the genes involved in N metabolism, relative abundance of bacteria, and soil chemical properties among different treatments. Spearman’s correlation coefficient and significance were calculated using the rcorr function in the R package “Hmisc” (v. 4.0-3). Bray-Curtis distance-based redundancy analysis (db-RDA) was used to examine the relationship between microbial community structure and soil chemical factors. Spearman’s correlation coefficient and its significance were calculated using the rcorr function in the R package “Hmisc” (v. 4.0-3).

## References

[1] Simberloff D, Martin JL, Genovesi P, Maris V, Wardle DA, Aronson J, Courchamp F, Galil B, Garcia-Berthou E, Pascal M, Pysek P, Sousa R, Tabacchi E, Vila M. 2013. Impacts of biological invasions: what’s what and the way forward. Trends Ecol Evol 28:58–66. doi:10.1016/j.tree.2012.07.013. 22889499

[2] Kueffer C. 2017. Plant invasions in the Anthropocene. Science 358:724–725. doi:10.1126/science.aao6371. 29123053

[3] Callaway RM, Ridenour WM. 2004. Novel weapons: invasive success and the evolution of increased competitive ability. Front Ecol Environ 2:436–443. doi:10.1890/1540-9295(2004)002[0436:NWISAT]2.0.CO;2.

[4] van der Putten WH. 2010. Impacts of soil microbial communities on exotic plant invasions. Trends Ecol Evol 25:512–519. doi:10.1016/j.tree.2010.06.006. 20638747

[5] Zhang F-J, Li Q, Chen F-X, Xu H-Y, Inderjit Wan F-H. 2017. Arbuscular mycorrhizal fungi facilitate growth and competitive ability of an exotic species *Flaveria bidentis*. Soil Biol Biochem 115:275–284. doi:10.1016/j.soilbio.2017.08.019.

[6] Chen X, Li Q, Wang LT, Meng YL, Jiao SN, Yin JL, Xu HY, Zhang FJ. 2021. Nitrogen uptake, not transfer of carbon and nitrogen by CMN, explains the effect of AMF on the competitive interactions between *Flaveria bidentis* and native species. Front Ecol Evol 9:1–11. doi:10.3389/fevo.2021.625519.

[7] Koyama A, Maherali H, Antunes PM, Pineda A. 2019. Plant geographic origin and phylogeny as potential drivers of community structure in root–inhabiting fungi. J Ecol 107:1720–1736. doi:10.1111/1365-2745.13143.

[8] Zhang C, Wang J, Liu GB, Song ZL, Fang LC. 2019. Impact of soil leachate on microbial biomass and diversity affected by plant diversity. Plant Soil 439:505–523. doi:10.1007/s11104-019-04032-x.

[9] Fierer N, Schimel JP, Holden PA. 2003. Variations in microbial community composition through two soil depth profiles. Soil Biol Biochem 35:167–176. doi:10.1016/S0038-0717(02)00251-1.

[10] Delgado-Baquerizo M, Reith F, Dennis PG, Hamonts K, Powell JR, Young A, Singh BK, Bissett A. 2018. Ecological drivers of soil microbial diversity and soil biological networks in the Southern Hemisphere. Ecology 99:583–596. doi:10.1002/ecy.2137. 29315530

[11] Soininen J, McDonald R, Hillebrand H. 2007. The distance decay of similarity in ecological communities. Ecography 30:3–12. doi:10.1111/j.0906-7590.2007.04817.x.

[12] Mitchell A, Romano GH, Groisman B, Yona A, Dekel E, Kupiec M, Dahan O, Pilpel Y. 2009. Adaptive prediction of environmental changes by microorganisms. Nature 460:220–224. doi:10.1038/nature08112. 19536156

[13] Lei YB, Xiao HF, Long FY. 2010. Impacts of alien plant invasions on biodiversity and evolutionary responses of native species. Biodiversity Sci 18:622–630. doi:10.3724/SP.J.1003.2010.622.

[14] Meisner A, Gera Hol WH, de Boer W, Krumins JA, Wardle DA, van der Putten WH. 2014. Plant–soil feedbacks of exotic plant species across life forms: a meta-analysis. Biol Invasions 16:2551–2561. doi:10.1007/s10530-014-0685-2.

[15] Zhang P, Li B, Wu JH, Hu SJ. 2019. Invasive plants differentially affect soil biota through litter and rhizosphere pathways: a meta-analysis. Ecol Lett 22:200–210. doi:10.1111/ele.13181. 30460738

[16] Xie Y, Li Z. 2001. Invasive species in China–an overview. Biodiversity Conservation 10:1317–1341. doi:10.1023/A:1016695609745.

[17] Xu HG, Qiang S, Han ZM, Guo JY, Huang ZG, Sun HY, He SP, Ding H, Wu HR, Wan FH. 2004. The distribution and introduction pathway of alien invasive species in China. Biodiversity Sci 12:626–638. doi:10.17520/biods.2004078.

[18] Ozaslan C, Onen H, Farooq S, Gunal H, Akyol N. 2016. Common ragweed: an emerging threat for sunflower production and human health in Turkey. Weed Biol Manage 16:42–55. doi:10.1111/wbm.12093.

[19] Zhang FJ, Li Q, Yerger EH, Chen X, Shi Q, Wan FH. 2018. AM fungi facilitate the competitive growth of two invasive plant species, *Ambrosia artemisiifolia* and *Bidens pilosa*. Mycorrhiza 28:703–715. doi:10.1007/s00572-018-0866-4. 30220052

[20] Johansen RB, Johnston P, Mieczkowski P, Perry GLW, Robeson MS, Vilgalys R, Burns BR. 2017. Scattered far and wide: a broadly distributed temperate dune grass finds familiar fungal root associates in its invasive range. Soil Biol Biochem 112:177–190. doi:10.1016/j.soilbio.2017.05.007.

[21] Rodriguez-Caballero G, Roldan A, Caravaca F. 2020. Invasive *Nicotiana glauca* shifts the soil microbial community composition and functioning of harsh and disturbed semiarid Mediterranean environments. Biol Invasions 22:2923–2940. doi:10.1007/s10530-020-02299-1.

[22] Li Q, Wan FH, Zhao M. 2022. Distinct soil microbial communities under *Ageratina adenophora* invasions. Plant Biol (Stuttg) 24:430–439. doi:10.1111/plb.13387. 35050505

[23] Luo LY, Jin DC, Zuo H, Zhang Z, Tan XQ, Zhang DY, Lu XY, Liu Y. 2017. Effects of *Rhodopseudomonas palustris* PSB06 on pepper rhizosphere microbial community structure. Huan Jing Ke Xue 38:735–742. doi:10.13227/j.hjkx.201606059. 29964533

[24] Zhang L, Wang XT, Wang J, Liao LR, Lei SL, Liu GB, Zhang C. 2022. Alpine meadow degradation depresses soil nitrogen fixation by regulating plant functional groups and diazotrophic community composition. Plant Soil 473:319–335. doi:10.1007/s11104-021-05287-z.

[25] Palaniyandi SA, Yang SH, Zhang LX, Suh JW. 2013. Effects of actinobacteria on plant disease suppression and growth promotion. Appl Microbiol Biotechnol 97:9621–9636. doi:10.1007/s00253-013-5206-1. 24092003

[26] Sreevidya M, Gopalakrishnan S, Kudapa H, Varshney RK. 2016. Exploring plant growth-promotion actinomycetes from vermicompost and rhizosphere soil for yield enhancement in chickpea. Braz J Microbiol 47:85–95. doi:10.1016/j.bjm.2015.11.030. 26887230 PMC4822753

[27] Peix A, Mateos PF, Rodriguez-Barrueco C, Martinez-Molina E, Velazquez E. 2001. Growth promotion of common bean (*Phaseolus vulgaris* L.) by a strain of *Burkholderia cepacia* under growth chamber conditions. Soil Biol Biochem 33:1927–1935. doi:10.1016/S0038-0717(01)00119-5.

[28] Santos AV, Dillon RJ, Dillon VM, Reynolds SE, Samuels RI. 2004. Ocurrence of the antibiotic producing bacterium *Burkholderia* sp. in colonies of the leaf-cutting ant *Atta sexdens rubropilosa*. FEMS Microbiol Lett 239:319–323. doi:10.1016/j.femsle.2004.09.005. 15476982

[29] Sun W, Shahrajabian MH, Cheng QI. 2021. Nitrogen fixation and diazotrophs–a review. Rom Biotechnol Lett 26:2834–2845. doi:10.25083/rbl/26.4/2834-2845.

[30] Zhang Y, Cao CL, Li RP, Jiang JH. 2022. Recent advance on the genus *Saccharothrix*. Acta Microbiol Sin 62:1600–1612. doi:10.13343/j.cnki.wsxb.20210580.

[31] Wang PP, Yang LF, Sun JL, Yang Y, Qu Y, Wang CX, Liu DQ, Huang LQ, Cui XM, Liu Y. 2021. Structure and function of rhizosphere soil and root endophytic microbial communities associated with root rot of *Panax notoginseng*. Front Plant Sci 12:752683. doi:10.3389/fpls.2021.752683. 35069616 PMC8766989

[32] Berg G, Rybakova D, Grube M, Koberl M. 2016. The plant microbiome explored: implications for experimental botany. J Exp Bot 67:995–1002. doi:10.1093/jxb/erv466. 26547794 PMC5395086

[33] Nuccio EE, Anderson-Furgeson J, Estera KY, Pett-Ridge J, De Valpine P, Brodie EL, Firestone MK. 2016. Climate and edaphic controllers influence rhizosphere community assembly for a wild annual grass. Ecology 97:1307–1318. doi:10.1890/15-0882.1. 27349106

[34] Cao M, Cui L, Sun H, Zhang X, Zheng X, Jiang J. 2021. Effects of *Spartina alterniflora* invasion on soil microbial community structure and ecological functions. Microorganisms 9:138. doi:10.3390/microorganisms9010138. 33435501 PMC7827921

[35] Novoa A, Rodríguez R, Richardson D, González L. 2014. Soil quality: a key factor in understanding plant invasion? The case of *Carpobrotus edulis* (L.) N.E.Br. Biol Invasions 16:429–443. doi:10.1007/s10530-013-0531-y.

[36] Gibbons SM, Lekberg Y, Mummey DL, Sangwan N, Ramsey PW, Gilbert JA. 2017. Invasive plants rapidly reshape soil properties in a grassland ecosystem. mSystems 2:e00178-16. doi:10.1128/mSystems.00178-16. 28289729 PMC5340861

[37] Rousk J, Baath E, Brookes PC, Lauber CL, Lozupone C, Caporaso JG, Knight R, Fierer N. 2010. Soil bacterial and fungal communities across a pH gradient in an arable soil. ISME J 4:1340–1351. doi:10.1038/ismej.2010.58. 20445636

[38] Wolters V, Joergensen RG. 1991. Microbial carbon turnover in beech forest soils at different stages of acidification. Soil Biol Biochem 23:897–902. doi:10.1016/0038-0717(91)90103-Q.

[39] Wang MP, Chen L, Zhang ZJ, Wang XJ, Qin S, Yan PS. 2017. Screening of alginate lyase-excreting microorganisms from the surface of brown algae. AMB Express 7:74. doi:10.1186/s13568-017-0361-x. 28374344 PMC5378567

[40] Fan K, Weisenhorn P, Gilbert JA, Shi Y, Bai Y, Chu H. 2018. Soil pH correlates with the co-occurrence and assemblage process of diazotrophic communities in rhizosphere and bulk soils of wheat fields. Soil Biol Biochem 121:185–192. doi:10.1016/j.soilbio.2018.03.017.

[41] Mollah S. 2004. The physics of the non-oxide perovskite superconductor MgCNi_3_. J Phys Condens Mat 16:1237–1276. doi:10.1088/0953-8984/16/43/R01.

[42] Green PT, O'Dowd DJ, Abbott KL, Jeffery M, Retallick K, Mac Nally R. 2011. Invasional meltdown: invader–invader mutualism facilitates a secondary invasion. Ecology 92:1758–1768. doi:10.1890/11-0050.1. 21939072

[43] Kuebbing SE, Classen AT, Simberloff D, Kardol P. 2014. Two co-occurring invasive woody shrubs alter soil properties and promote subdominant invasive species. J Appl Ecol 51:124–133. doi:10.1111/1365-2664.12161.

[44] Liu XW, He FL, Qi CM, Quan QG, Ao Y, Li Y, Luo Y, Yan DD, Cao AC. 2016. Effect of invasive plants *Ambrosia artemisiifolia* L. on soil carbon and nitrogen transition. Acta Agriculturae Zhejiangensis 28:197–301. doi:10.3969/j.issn.1004-1524.2016.02.19.

[45] Zhao TQ, Zhang K, Zheng H, Chen FL, Lin XQ. 2011. Pathways of exotic plant impacts on nitrogen cycling in terrestrial ecosystem. Ecol Sci 30:207–212. doi:10.3969/j.issn.1008-8873.2011.02.020.

[46] Wang XL, Cui D, Yang HJ, Yan JJ, Cao WQ, Liu HJ, Zhang J, Sha WL. 2021. Effects of *Sophora alopecuroides* invasion on soil nutrients and active organic carbon components at different altitudes in Yili valley steppe. Environ Chem 4:1232–1242. doi:10.7524/j.issn.0254-6108.2019120102.

[47] Xu H, Hu CC, Xu SQ, Sun XC, Liu XY. 2018. Effects of exotic plant invasion on soil nitrogen availability. Chinese J Plant Ecol 42:1120–1130. doi:10.17521/cjpe.2018.0219.

[48] Weng Z, Li GQ, Sale P, Tang CX. 2021. Application of calcium nitrate with phosphorus promotes rhizosphere alkalization in acid subsoil. Eur J Soil Sci 73:1–13. doi:10.1111/ejss.13153.

[49] Yu HW, He WM, Osborne B. 2021. Congeneric invasive versus native plants utilize similar inorganic nitrogen forms but have disparate use efficiencies. J Plant Ecol 14:180–190. doi:10.1093/jpe/rtaa085.

[50] Milbau A, Nijs I, Van Peer L, Reheul D, De Cauwer B. 2003. Disentangling invasiveness and invasibility during invasion in synthesized grassland communities. New Phytol 159:657–667. doi:10.1046/j.1469-8137.2003.00833.x. 33873590

[51] Xing TT, Cai AD, Lu CA, Ye HL, Wu HL, Huai SC, Wang JY, Xu MG, Lin QM. 2022. Increasing soil microbial biomass nitrogen in crop rotation systems by improving nitrogen resources under nitrogen application. J Integr Agric 21:1488–1500. doi:10.1016/S2095-3119(21)63673-0.

[52] Liao LR, Wang J, Zhang C, Liu GB, Song ZL. 2019. Effects of grazing exclusion on the abundance of functional genes involved in soil nitrogen cycling and nitrogen storage in semiarid grassland. Ying Yong Sheng Tai Xue Bao 30:3473–3481. doi:10.13287/j.1001-9332.201910.002. 31621234

[53] Ma XY, Jiang L, Song YY, Sun L, Song CC, Hou AX, Gao JL, Du Y. 2019. Effects of temperature and moisture changes on functional gene abundance of soil nitrogen cycle in permafrost peatland. Acta Ecol Sinica 41:6707–6717. doi:10.5846/stxb202008062048.

[54] Angeloni NL, Jankowski KJ, Tuchman NC, Kelly JJ. 2006. Effects of an invasive cattail species (*Typha* × *glauca*) on sediment nitrogen and microbial community composition in a freshwater wetland. FEMS Microbiol Lett 263:86–92. doi:10.1111/j.1574-6968.2006.00409.x. 16958855

[55] Zhang QF, Peng JJ, Chen Q, Li XF, Xu CY, Yin HB, Yu S. 2011. Impacts of *Spartina alterniflora* invasion on abundance and composition of ammonia oxidizers in estuarine sediment. J Soils Sediments 11:1020–1031. doi:10.1007/s11368-011-0369-9.

[56] Wasserstrom H, Steinberger Y. 2018. Soil microbial-community alteration in response to heterotheca subaxillaris–an invasive alien plant. Environ Natural Resource Res 8:85–99. doi:10.5539/enrr.v8n2p85.

[57] Chen BM, Peng SL, Ni GY. 2009. Effects of the invasive plant *Mikania micrantha* H.B.K. on soil nitrogen availability through allelopathy in South China. Biol Invasions 11:1291–1299. doi:10.1007/s10530-008-9336-9.

[58] Chen BM, Wei HJ, Chen WB, Zhu ZC, Yuan YR, Zhang YL, Lan ZG, 1School of Life Sciences, Sun Yat-Sen University, State Key Laboratory of Biocontrol, Guangzhou 510275, China. 2018. Effects of plant invasion on soil nitrogen transformation processes and its associated microbial. Chinese J Plant Ecol 42:1071–1081. doi:10.17521/cjpe.2018.0138.

[59] Huber PJ. 1981. Robust statistics. John Wiley & Sons, New York.

[60] Tang H, Xiao X, Xu Y, Li C, Cheng K, Pan X, Li W. 2019. Utilization of carbon sources in the rice rhizosphere and non-rhizosphere soils with different long-term fertilization management. J Basic Microbiol 59:621–631. doi:10.1002/jobm.201800736. 30980731

[61] Lu RK. 1999. Soil agrochemical analysis method. China Agricultural Science and Technology Press, Beijing.

[62] Du EW, Chen X, Li Q, Chen FX, Xu HY, Zhang FJ. 2020. *Rhizoglomus intraradices* and associated *Brevibacterium frigoritolerans* enhance the competitive growth of *Flaveria bidentis*. Plant Soil 453:281–295. doi:10.1007/s11104-020-04594-1.

[63] Bao SD. 2000. Agrochemical analysis of soil. Chinese Agricultural Press, Beijing, china.

[64] Nelson DW, Sommers LE. 1972. A simple digestion procedure for estimation of total nitrogen in soils and sediments. J Environ Qual 1:423–425. doi:10.2134/jeq1972.00472425000100040020x.

