# Supplementary material for: Nitrate Nitrogen and pH Correlate with Changes in Rhizosphere Microbial Community Assemblages during Invasion of Ambrosia artemisiifolia and Bidens pilosa
Source: Microbiol Spectr. 2022 Dec 13;11(1):e03649-22. doi: 10.1128/spectrum.03649-22 (PMC9927480; doi:10.1128/spectrum.03649-22)
Supplement: Supplemental file 1 — Supplemental material. Download spectrum.03649-22-s0001.pdf, PDF file, 2.2 MB [file spectrum.03649-22-s0001.pdf]

Table S1 Comparative analysis of relative abundance of bacteria in rhizosphere soil of two invasive and three native plant species in different treatments

	treatments	Relative abundance	treatments	Relative abundance
1	A	0.981±0.001a	B	0.981±0.001a
	AC_a	0.965±0.003c	BC_b	0.971±0.001bc
	AC_c	0.966±0.003c	BC_c	0.967±0.004c
	C	0.974±0.001b	C	0.972±0.001bc
	CK	0.974±0.001b	CK	0.974±0.001b
2	A	0.981±0.001a	B	0.981±0.001a
	AM_a	0.964±0.003c	BM_b	0.966±0.002c
	AM_m	0.96±0.002c	BM_m	0.967±0.003c
	M	0.963±0.001c	M	0.963±0.001c
	CK	0.974±0.001b	CK	0.974±0.001b
3	A	0.981±0.001a	B	0.981±0.001a
	AS_a	0.968±0.002c	BS_b	0.968±0.002bc
	AS_s	0.969±0.002c	BS_s	0.967±0.003bc
	S	0.966±0.003c	S	0.965±0.003c
	CK	0.974±0.001b	CK	0.974±0.001b

Note: The values are the mean ± standard deviation, and different lowercase letters in the same column indicate significant differences at the 5% significance level; : A: *A. artemisiifolia* monoculture, C: *C. serotinum* monoculture, M: *M. suaveolens* monoculture, S, *S. viridis* monoculture, AC-a: *A. artemisiifolia* in the mixture between *A. artemisiifolia* and *C. serotinum*, AM-a, : *A. artemisiifolia* in the mixture between *A. artemisiifolia* and *M. suaveolens*, AS-a: *A. artemisiifolia* in the mixture between *A. artemisiifolia* and *S. viridis*, AC-c: *C. serotinum* in the mixture between *A. artemisiifolia* and *C. serotinum*, AM-m: *M. suaveolens* in the mixture between *A. artemisiifolia* and *M. suaveolens*, AS-s: *S. viridis* in the mixture between *A. artemisiifolia* and *S. viridis*, B: *B. pilosa* monoculture, BC-b: *B. pilosa* in the mixture between *B. pilosa* and *C. serotinum*, BM-b: *B. pilosa* in the mixture between *B. pilosa* and *M. suaveolens*, BS-b, *B. pilosa* in the mixture between *B. pilosa* and *S. viridis,* BC-c: *C. serotinum* in the mixture between *B. pilosa* and *C. serotinum*, BC-m: *M. suaveolens* in the mixture between *B. pilosa* and *M. suaveolens*, BS-s: *S. viridis* in the mixture between *B. pilosa* and *S. viridis*

Table S2 Comparative analysis of chemical properties in rhizosphere soil of *Ambrosia artemisiifolia* and three native plant species in different treatments

		TN（mg/kg）	TP（mg/kg）	AK（mg/kg）	AP（mg/kg）	OC (%)	NH_4^+-N（mg/kg）	N0$_3^-$-N（mg/kg）	pH
	A	1140.67±2.31b	852.00±8.72a	224.00±3.61c	5.02±0.34ab	0.95±0.02ab	4.93±0.04b	5.71±0.33e	8.65±0.01a
	AC_a	1162.33±7.51a	786.00±13.45c	233.33±0.58c	4.64±0.22b	0.98±0.02a	4.06±0.04c	11.13±0.06b	8.54±0.01c
1	AC_c	1146.50±5.50b	834.50±6.50a	248.50±2.50b	5.33±0.10a	0.95±0.01ab	4.54±0.09b	7.17±0.05d	8.59±0.01b
	C	1126.33±3.51c	828.00±7.55ab	290.67±7.09a	4.94±0.25ab	0.94±0.02ab	6.18±0.29a	17.37±0.12a	8.50±0.01cd
	CK	944.94±3.63d	800.63±15.40bc	228.99±6.21c	3.14±0.01c	0.92±0.03c	1.31±0.14d	9.50±0.16c	8.47±0.03d
	A	1140.67±2.31b	852.00±8.72a	224.01±3.61a	5.02±0.34a	0.95±0.02b	4.93±0.04a	5.71±0.33d	8.65±0.01a
	AM_a	1179.33±1.53a	859.00±1.73a	228.33±4.93a	5.32±0.28a	1.02±0.01a	4.68±0.32ab	17.83±0.67b	8.49±0.01d
2	AM_m	1148.33±0.58b	865.00±5.29a	193.00±4.58b	4.33±0.23b	0.96±0.02b	4.23±0.40b	10.13±0.06c	8.55±0.01c
	M	1106.67±6.66c	821.33±0.58b	134.67±2.08c	2.33±0.19d	0.89±0.01c	0.11±0.00d	26.57±0.47a	8.62±0.01b
	CK	944.94±3.63d	800.63±15.40b	228.99±6.21a	3.14±0.01c	0.92±0.03bc	1.31±0.14c	9.50±0.16c	8.47±0.03d
	A	1140.67±2.31c	852.00±8.72c	224.00±3.61d	5.02±0.34c	0.95±0.02bc	4.93±0.04b	5.71±0.33e	8.65±0.01a
	AS_a	1188.00±2.65b	880.67±5.86b	292.67±6.51c	5.33±0.13c	1.00±0.02b	5.03±0.00b	6.31±0.14d	8.51±0.01d
3	AS_s	1236.67±3.06a	825.33±6.51d	323.67±1.16b	6.48±0.37b	1.12±0.02a	4.12±0.01c	7.81±0.01b	8.41±0.01e
	S	1177.00±8.19b	916.00±4.36a	456.67±5.69a	11.57±0.16a	1.00±0.01b	7.02±0.33a	7.30±0.12c	8.61±0.01b
	CK	944.94±3.63d	800.63±15.40e	228.99±6.21d	3.14±0.01d	0.92±0.03c	1.31±0.14d	9.50±0.16a	8.47±0.03c
	AC_a	1162.33±7.51b	786.00±13.45b	233.33±0.58b	4.64±0.22b	0.98±0.02a	4.06±0.04c	11.13±0.06b	8.54±0.01a
4	AM_a	1179.33±1.53a	859.00±1.73a	228.33±4.93b	5.32±0.28a	1.02±0.01a	4.68±0.32b	17.83±0.67a	8.49±0.01c
	AS_a	1188.00±2.65a	880.67±5.86a	292.67±6.51a	5.33±0.13a	1.00±0.02a	5.03±0.00a	7.80±0.14c	8.51±0.01b
	C	1126.33±3.51b	828.00±7.55b	290.67±7.09b	4.94±0.25b	0.94±0.02b	6.18±0.29b	17.37±0.12b	8.50±0.01b
5	M	1106.67±6.66c	821.33±0.58b	134.67±2.08c	2.33±0.19c	0.89±0.01c	0.11±0.00c	26.57±0.47a	8.62±0.01a
	S	1177.00±8.19a	916.00±4.36a	456.67±5.69a	11.57±0.16a	1.00±0.01a	7.02±0.33a	7.30±0.12c	8.61±0.01a

Note: The values are the mean ± standard deviation, and different lowercase letters in the same column indicate significant differences at the 5% significance level; TN: total nitrogen, TP: total phosphorus, AK: available potassium, AP: effective Phosphorus, OC: organic carbon, NH^{4+}-N: ammonium nitrogen, NO^{3-}-N: nitrate nitrogen. A: *A. artemisiifolia* monoculture, C: *C. serotinum* monoculture, M: *M. suaveolens* monoculture, S, *S. viridis* monoculture, AC-a: *A. artemisiifolia* in the mixture between *A. artemisiifolia* and *C. serotinum*, AM-a, : *A. artemisiifolia* in the mixture between *A. artemisiifolia* and *M. suaveolens*, AS-a: *A. artemisiifolia* in the mixture between *A. artemisiifolia* and *S. viridis*, AC-c: *C. serotinum* in the mixture between *A. artemisiifolia* and *C. serotinum*, AM-m: *M. suaveolens* in the mixture between *A. artemisiifolia* and *M. suaveolens*, AS-s: *S. viridis* in the mixture between *A. artemisiifolia* and *S. viridis*

Table S3 Comparative analysis of chemical properties of rhizosphere soil of *Bidens pilisa* and three native plant species in different treatments

		TN（mg/kg）	TP（mg/kg）	AK（mg/kg）	AP（mg/kg）	OC（%）	NH_4^+-N（mg/kg）	$N0_3^-$-N（mg/kg）	pH
	B	1238.17±3.01a	850.83±3.00ab	176.67±5.50d	6.41±0.31a	1.12±0.02a	5.86±0.33a	6.17±0.05e	8.64±0.02a
	BC_b	1277.83±13.01a	889.50±14.73a	235.17±1.00b	5.80±0.25a	1.09±0.01a	4.43±0.23b	7.15±0.39d	8.51±0.01b
1	BC_c	1221.33±11.50a	844.00±12.00ab	217.33±0.50c	4.82±0.20b	1.10±0.04a	4.33±0.20b	8.17±0.19c	8.65±0.01a
	C	1126.33±3.51b	828.00±7.50b	290.67±7.00a	4.94±0.25b	0.94±0.02b	6.18±0.27a	17.37±0.10a	8.50±0.01b
	CK	944.94±3.63c	800.63±15.40b	228.99±6.21bc	3.14±0.01c	0.92±0.02b	1.31±0.14c	9.50±0.16b	8.47±0.03b
	B	1238.17±3.01b	850.83±2.75b	176.67±5.03b	6.41±0.22a	1.12±0.02b	5.86±0.33a	6.17±0.05e	8.64±0.02a
	BM_b	1323.33±11.08a	934.50±12.50a	187.50±3.77b	5.89±0.19a	1.15±0.02b	5.53±0.06a	7.98±0.31d	8.53±0.01c
2	BM_m	1241.50±10.10b	835.00±13.00b	157.00±2.00c	4.42±0.00b	1.24±0.05a	5.63±0.00a	8.79±0.01c	8.51±0.01bc
	M	1106.67±6.66c	821.33±0.58bc	134.67±2.08d	2.33±0.19d	0.89±0.01c	0.11±0.00c	26.57±0.47a	8.62±0.01b
	CK	944.94±3.63d	800.63±15.40c	228.99±6.21ab	3.14±0.01c	0.92±0.03bc	1.31±0.14b	9.50±0.16b	8.47±0.03d
	B	1238.17±3.01b	850. 83±2. 75b	176. 67±5. 03d	6. 41±0. 22d	1. 12±0. 02a	5. 86±0. 33bc	6.17±0.05e	8. 64±0. 02a
	BS_b	1292.17±5.34a	855.50±8.22b	222. 83±5. 01c	7.53±0.30c	1. 16±0. 01a	6. 34±0. 13ab	8.53±0.09b	8.56±0.00c
3	BS_s	1225. 33±6. 66b	813.17±10.40bc	278.50±0.50b	10.40±0.30b	1. 16±0. 05a	5. 12±0. 38c	7.63±0.14c	8.49±0.00d
	S	1177.00±8.19c	916.00±4.36a	456.67±5.69a	11.57±0.16a	1.00±0.01b	7.02±0.33a	7.30±0.12d	8.61±0.01b
	CK	944.94±3.63d	800.63±15.40c	228.99±6.21bc	3.14±0.01e	0.92±0.03bc	1.31±0.14d	9.50±0.16a	8.47±0.03d
	BC_b	1277.83±13.01a	889.50±14.73ab	235.17±1.04a	5.80±0.23b	1.09±0.01b	4.43±0.22c	7.15±0.39b	8.51±0.01b
4	BM_b	1323.33±8.08a	934.50±7.70a	187.50±3.77b	5.89±0.19b	1.15±0.02a	5.53±0.06b	7.98±0.31a	8.53±0.01b
	BS_b	1292.17±5.34a	855.50±8.22b	222.83±5.01a	7.53±0.30a	1.16±0.01a	6.34±0.13a	8.53±0.09a	8.56±0.00a

Note: The values are mean ± standard deviation, and different lowercase letters in the same column indicate significant differences at the 5% significance level; TN: total nitrogen, TP: total phosphorus, AK: available potassium, AP: effective Phosphorus, OC: organic carbon, NH^{4+}-N: ammonium nitrogen, NO^{3-}-N: nitrate nitrogen. B: *B. pilosa* monoculture, BC-b: *B. pilosa* in the mixture between *B. pilosa* and *C. serotinum*, BM-b: *B. pilosa* in the mixture between *B. pilosa* and *M. suaveolens*, BS-b, *B. pilosa* in the mixture between *B. pilosa* and *S. viridis,* BC-c: *C. serotinum* in the mixture between *B. pilosa* and *C. serotinum*, BC-m: *M. suaveolens* in the mixture between *B. pilosa* and *M. suaveolens*, BS-s: *S. viridis* in the mixture between *B. pilosa* and *S. viridis*

Table S4: Determination coefficient of soil physicochemical factors affecting soil microorganisms by db-RDA analysis

		r^2	p_values		r^2	p_values
TN	AC	0.04	0.80	BC	0.11	0.60
TP		0.50	0.03		0.02	0.90
AK		0.41	0.08		0.75	0.01
AP		0.47	0.07		0.48	0.07
OC		0.04	0.84		0.42	0.08
NH_4^+-N		0.15	0.47		0.42	0.12
$N0_3^-$-N		0.82	0.01		0.74	0.04
PH		0.95	0.01		0.95	0.00
TN	AM	0.14	0.49	BM	0.27	0.28
TP		0.46	0.05		0.01	0.96
AK		0.26	0.25		0.22	0.34
AP		0.23	0.33		0.49	0.05
OC		0.10	0.61		0.82	0.00
NH_4^+-N		0.42	0.06		0.71	0.01
$N0_3^-$-N		0.79	0.00		0.67	0.01
PH		0.57	0.02		0.86	0.00
TN	AS	0.65	0.01	AS	0.06	0.73
TP		0.12	0.57		0.14	0.52
AK		0.44	0.06		0.37	0.14
AP		0.19	0.39		0.50	0.04
OC		0.60	0.01		0.08	0.67
NH_4^+-N		0.05	0.80		0.23	0.32
$N0_3^-$-N		0.69	0.00		0.54	0.04
PH		0.53	0.04		0.68	0.04

Note: The values in the table are the mean ± standard deviation, and different lowercase letters in the same column indicate significant differences at the 5% significance level; TN: total nitrogen, TP: total phosphorus, AK: available potassium, AP: effective Phosphorus, OC: organic carbon, NH^{4+}-N: ammonium nitrogen, NO^{3-}-N: nitrate nitrogen, r^2: degree of correlation, p_values: Significance. AC, treatments between *A. artemisiifolia* and *C. serotinum*; AM, treatments between *A. artemisiifolia* and *M. suaveolen*; AS, treatments between *A. artemisiifolia* and *S. viridis*; BC, treatments between *B. pilisa* and *C.* serotinum; BM, treatments between *B. pilisa* and *M.* suaveolen; BS, treatments between *B. pilisa* and *S. viridis.*

Table S5 The climatic environment from 2012 to 2019

Year	Annual average temperature (°C)	Annual average humidity (%)	annual rainfall (mm)
2012	11.31	57.21	609.85
2013	11.46	61.43	261.37
2014	12.71	58.14	357.13
2015	12.71	58.48	418.60
2016	12.55	58.35	228.60
2017	14.02	50.13	98.80
2018	12.24	56.01	598.99
2019	12.78	55.98	441.00

Table S6 Treatments and soil samples in the long-term experiment

Treatments		Soil sample from the rhizosphere soil of plant species in different treatment	
Full name	Abbreviations	Full name	abbreviations
monoculture of *A. artemisiifolia*	A	*A. artemisiifolia* in monoculture of *A. artemisiifolia*	A
mixture of *A. artemisiifolia* and *C. serotinum*	AC	*A. artemisiifolia* in mixture of *A. artemisiifolia* and *C. serotinum*	AC-a
		C. serotinum in mixture of *A. artemisiifolia* and *C. serotinum*	AC-c
mixture of *A. artemisiifolia* and *M. suaveolens*	AM	*A. artemisiifolia* in mixture of *A. artemisiifolia* and *M. suaveolens*	AM-a
		M. suaveolens in mixture of *A. artemisiifolia* and *M. suaveolens*	AM-m
mixture of *A. artemisiifolia* and *S. viridis*	AS	*A. artemisiifolia* in mixture of *A. artemisiifolia* and *S. viridis*	AS-a
		S. viridis in mixture of *A. artemisiifolia* and *S. viridis*	AS-s
monoculture of *B. pilosa*	B	*B. pilosa* in monoculture of *B. pilosa*	B
mixture of *B. pilosa* and *C. serotinum*	BC	*B. pilosa* in mixture of *B. pilosa* and *C. serotinum*	BC-b
		C. serotinum in mixture of *B. pilosa* and *C. serotinum*	BC-c
mixture of *B. pilosa* and *M. suaveolens*	BM	*B. pilosa* in mixture of *B. pilosa* and *M. suaveolens*	BM-b
		M. suaveolens in mixture of *B. pilosa* and *M. suaveolens*	BM-m
mixture of *B. pilosa* and *S. viridis*	BS	*B. pilosa* in mixture of *B. pilosa* and *S. viridis*	BS-b
		S. viridis in mixture of *B. pilosa* and *S. viridis*	BS-s
monoculture of *C. serotinum*	C	*C. serotinum* in monoculture of *C. serotinum*	C
monoculture of *M. suaveolens*	M	*M. suaveolens* in monoculture of *M. suaveolens*	M
monoculture of *S. viridis*	S	From the rhizosphere soil of *S. viridis* in monoculture of *S. viridis*	S
control	CK	No plant	CK

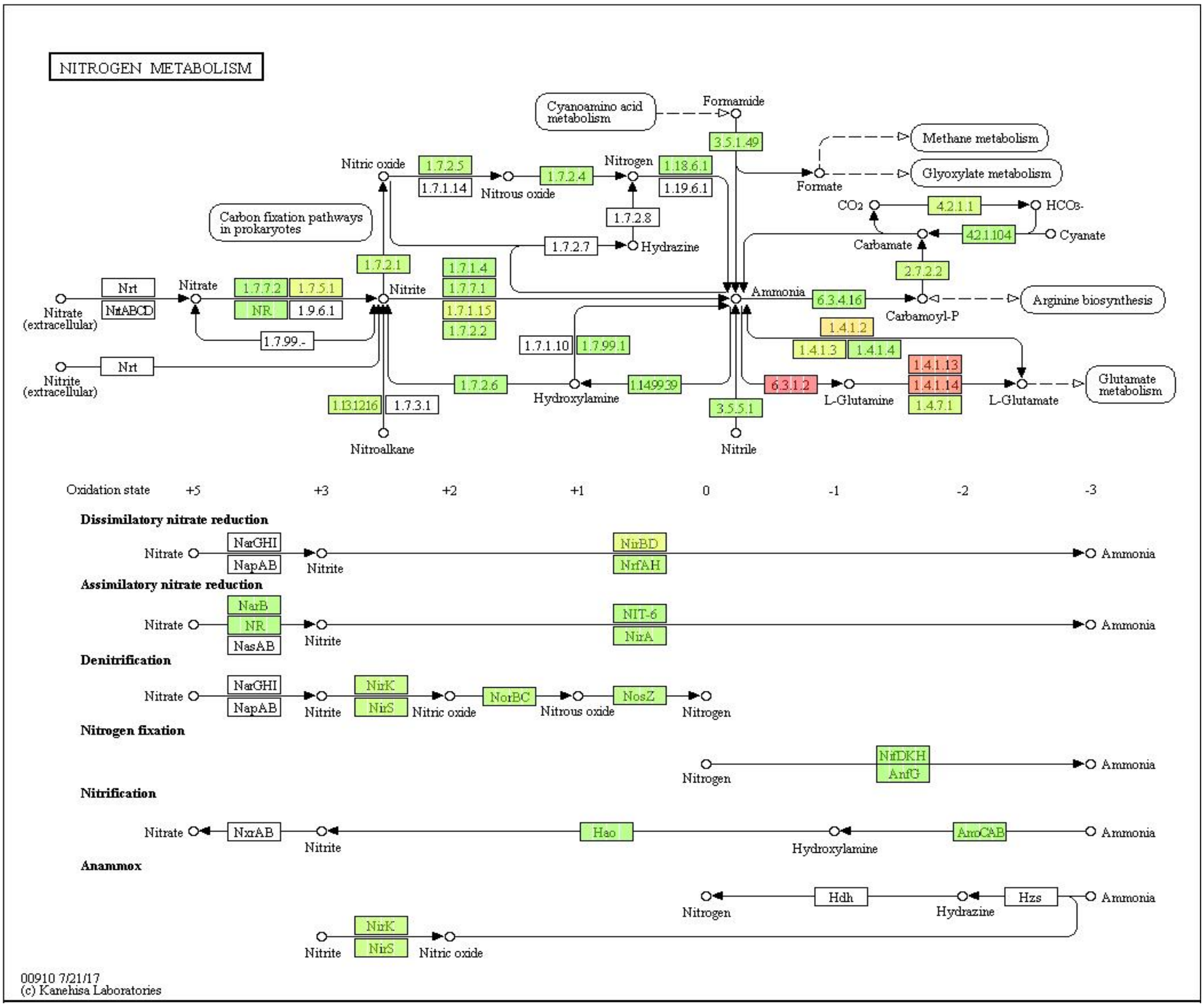

FIG S1 Microbial nitrogen metabolism in the rhizosphere soil of two invasive and three native plants under different treatments

Notes: The filled box in the figure represents the functional enzyme genes related to nitrogen metabolism. The different color depth indicates the difference in the relative abundance of functional enzyme genes. Red indicates the highest relative abundance, followed by orange. Green indicates the lowest relative abundance.

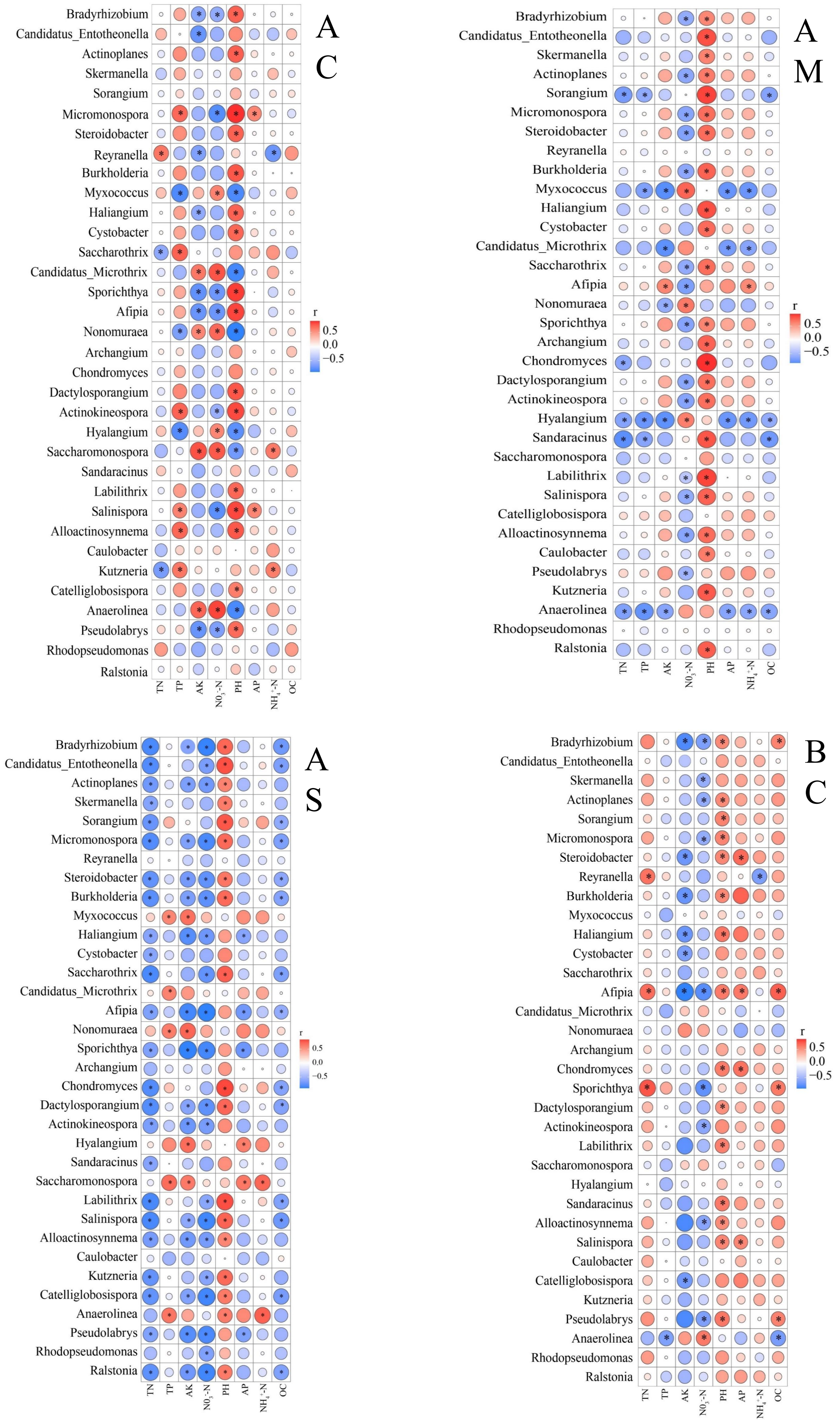
A
C
Bradyrhizobium
Candidatus_Entotheonella
Actinoplanes
Skermanella
Sorangium
Micromonospora
Steroidobacter
Reyranella
Burkholderia
Myxococcus
Haliangium
Cystobacter
Saccharothrix
Candidatus_Microthrix
Sporichthya
Afipia
Nonomuraea
Archangium
Chondromyces
Dactylosporangium
Actinokineospora
Hyalangium
Saccharomonospora
Sandaracinus
Labilithrix
Salinispora
Alloactinosynnema
Caulobacter
Kutzneria
Catelliglobosispora
Anaerolinea
Pseudolabrys
Rhodopseudomonas
Ralstonia
TN TP AK NO_3^--N PH AP NH_4^+-N OC
r
0.5
0.0
−0.5
A
M
Bradyrhizobium
Candidatus_Entotheonella
Skermanella
Actinoplanes
Sorangium
Micromonospora
Steroidobacter
Reyranella
Burkholderia
Myxococcus
Haliangium
Cystobacter
Candidatus_Microthrix
Saccharothrix
Afipia
Nonomuraea
Sporichthya
Archangium
Chondromyces
Dactylosporangium
Actinokineospora
Hyalangium
Sandaracinus
Saccharomonospora
Labilithrix
Salinispora
Catelliglobosispora
Alloactinosynnema
Caulobacter
Pseudolabrys
Kutzneria
Anaerolinea
Rhodopseudomonas
Ralstonia
TN TP AK NO_3^--N PH AP NH_4^+-N OC
r
0.5
0.0
−0.5
A
S
Bradyrhizobium
Candidatus_Entotheonella
Actinoplanes
Skermanella
Sorangium
Micromonospora
Reyranella
Steroidobacter
Burkholderia
Myxococcus
Haliangium
Cystobacter
Saccharothrix
Candidatus_Microthrix
Afipia
Nonomuraea
Sporichthya
Archangium
Chondromyces
Dactylosporangium
Actinokineospora
Hyalangium
Sandaracinus
Saccharomonospora
Labilithrix
Salinispora
Alloactinosynnema
Caulobacter
Kutzneria
Catelliglobosispora
Anaerolinea
Pseudolabrys
Rhodopseudomonas
Ralstonia
TN TP AK NO_3^--N PH AP NH_4^+-N OC
r
0.5
0.0
−0.5
B
C
Bradyrhizobium
Candidatus_Entotheonella
Skermanella
Actinoplanes
Sorangium
Micromonospora
Steroidobacter
Reyranella
Burkholderia
Myxococcus
Haliangium
Cystobacter
Saccharothrix
Afipia
Candidatus_Microthrix
Nonomuraea
Archangium
Chondromyces
Sporichthya
Dactylosporangium
Actinokineospora
Labilithrix
Saccharomonospora
Hyalangium
Sandaracinus
Alloactinosynnema
Salinispora
Caulobacter
Catelliglobosispora
Kutzneria
Pseudolabrys
Anaerolinea
Rhodopseudomonas
Ralstonia
TN TP AK NO_3^--N PH AP NH_4^+-N OC
r
0.5
0.0
−0.5

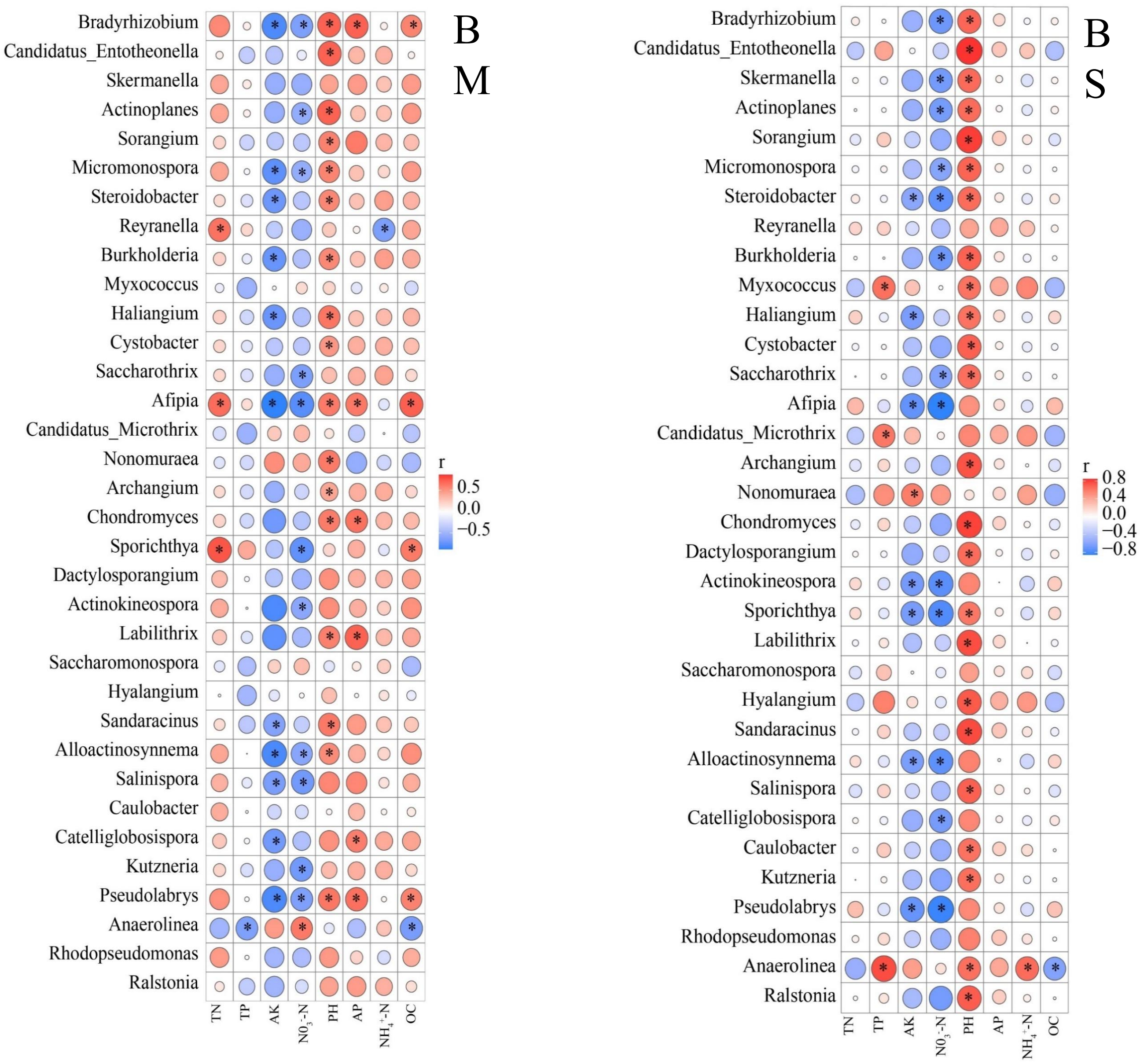

FIG. S2 Heatmap diagram of the correlation analysis of genera clustered in the rhizosphere soil of two invasive species with chemical factors in different treatments

Notes: AC, treatments between *A. artemisiifolia* and *C. album*; AM, treatments between *A. artemisiifolia* and *M. officinalis*; AS, treatments between *A. artemisiifolia* and *S. viridis*; BC, treatments between *B. pilosa* and *C. album*; BM, treatments between *B. pilosa* and *M. officinalis*; BS, treatments between *B. pilosa* and *S. viridis.* The circle size in the figure indicates the degree of correlation between soil microbe and chemical factors, red indicates the correlation is positive, blue indicates the correlation is negative. The asterisk indicates that the correlation between soil microbe and chemical factor is significant.
